# Supplementary material for: Allosteric Binding Sites On Nuclear Receptors: Focus On Drug Efficacy and Selectivity
Source: Int J Mol Sci. 2020 Jan 14;21(2):534. doi: 10.3390/ijms21020534 (PMC7014104; doi:10.3390/ijms21020534)
Supplement: Supplementary file 1 [file ijms-21-00534-s001.pdf]

Supporting Information

Allosteric Binding Sites On Nuclear Receptors: Focus On Drug Efficacy and Selectivity

André Fischer and Martin Smieško*

Computational Pharmacy Group, Department of Pharmaceutical Sciences, Klingelbergstrasse 50, 4056 Basel

Corresponding author contact: martin.smiesko@unibas.ch

Abstract: Nuclear receptors (NRs) are highly relevant drug targets in major indications such as oncologic, metabolic, reproductive and immunologic diseases. However, currently marketed drugs designed towards the orthosteric binding site of NRs often suffer from resistance mechanisms and poor selectivity. The identification of two superficial allosteric sites activation function-2 (AF-2) and binding function-3 (BF-3) as novel drug targets sparked the development of inhibitors, while selectivity concerns due to a high conservation degree remained. To determine important pharmacophores and hydration sites among AF-2 and BF-3 of eight hormonal NRs, we systematically analyzed over 10 µs of molecular dynamics simulations including simulations in explicit water and solvent mixtures. In addition, a library of over 300 allosteric inhibitors was evaluated by molecular docking. Based on our results, we suggest the BF-3 site to offer a higher potential for drug selectivity as opposed to the AF-2 site that is more conserved among the selected receptors. Detected similarities among the AF-2 sites of various NRs urge for a broader selectivity assessment in future studies. In combination with the supporting materials, this work provides a foundation to improve both selectivity and potency of allosteric inhibitors in a rational manner and increase the therapeutic applicability of this promising compound class.

Table of Contents

Supporting Results and Discussion

Sequence Similarity Among Hormonal NRs: Figures S1-S2 2
Distinct Pharmacophores of the Allosteric Sites: Figures S3-S4; Tables S1-S8 4
Conformational Change: Figures S5-S7 8
Hydration Sites of the Allosteric Sites: Figure S8-S10, Tables S9-S16 10
Molecular Docking: Figures S11-S18, Tables S17-S18 20

Supporting Materials and Methods

Sequence Alignment and Analysis: Figure S19, Table S19 24
Ligand Preparation: Table S20 25
Protein Preparation: Table S21 31
MD Simulations: Tables S22-S23 32
Crystal Structure Analysis: Table S24 32
SI References 32

Supporting Results and Discussion

Sequence Similarity Among Hormonal NRs

Figure S1. Residues and surface representation of AF-2 and BF-3 sites for AR, ERα, ERβ, and GR.

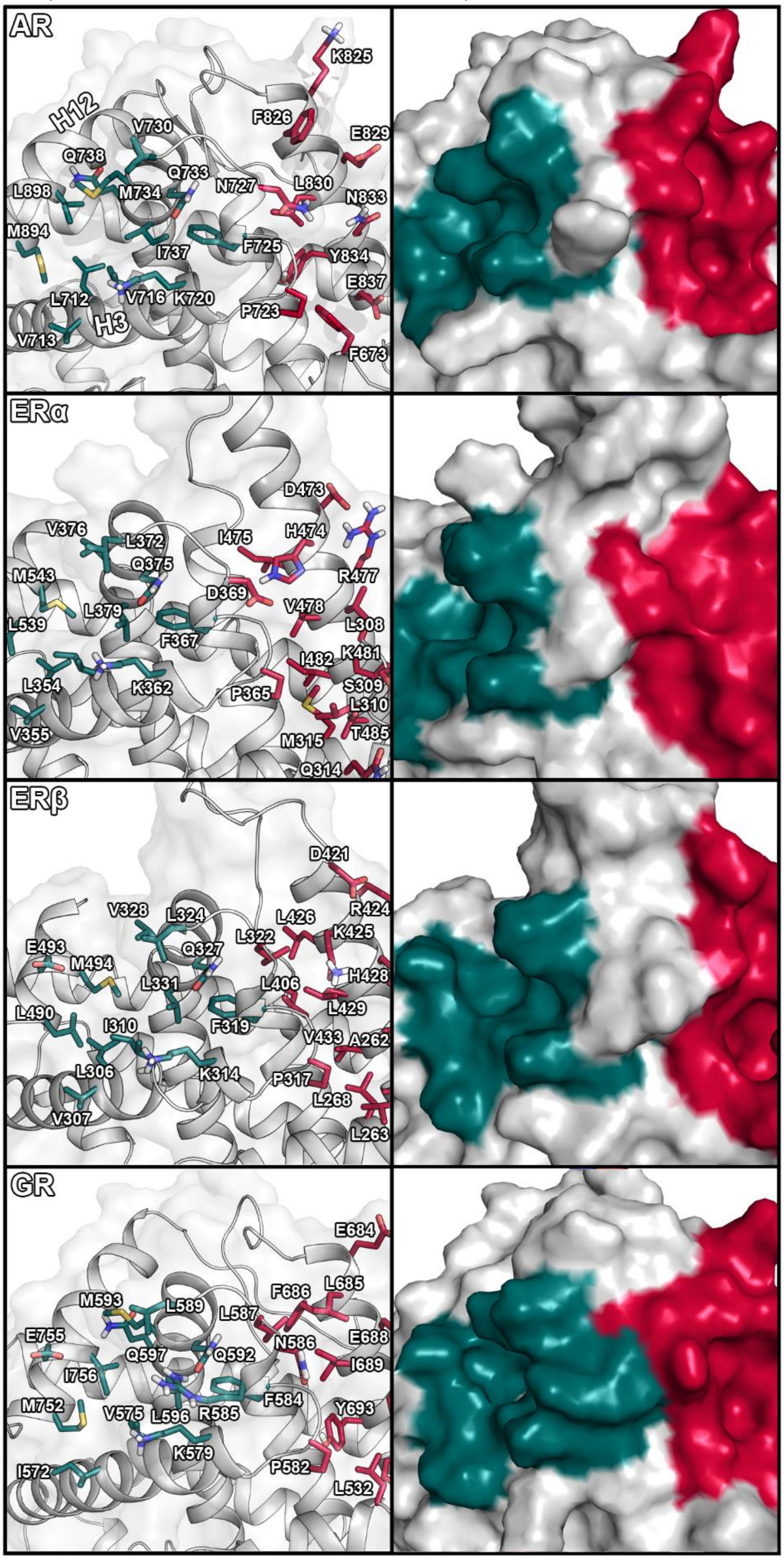

Representation of the AF-2 and BF-3 sites of AR, ERα, ERβ, and GR. The AF-2 is shown in pine green, while the BF-3 site was colored red. The surface was colored according to the type of residue (blue, positive charge; red, negative charge; green, non-polar; yellow, cysteine; purple, glycine; light blue, histidine).

Figure S2. Residues and surface representation of AF-2 and BF-3 sites for MR, PR, TRα, and TRβ.

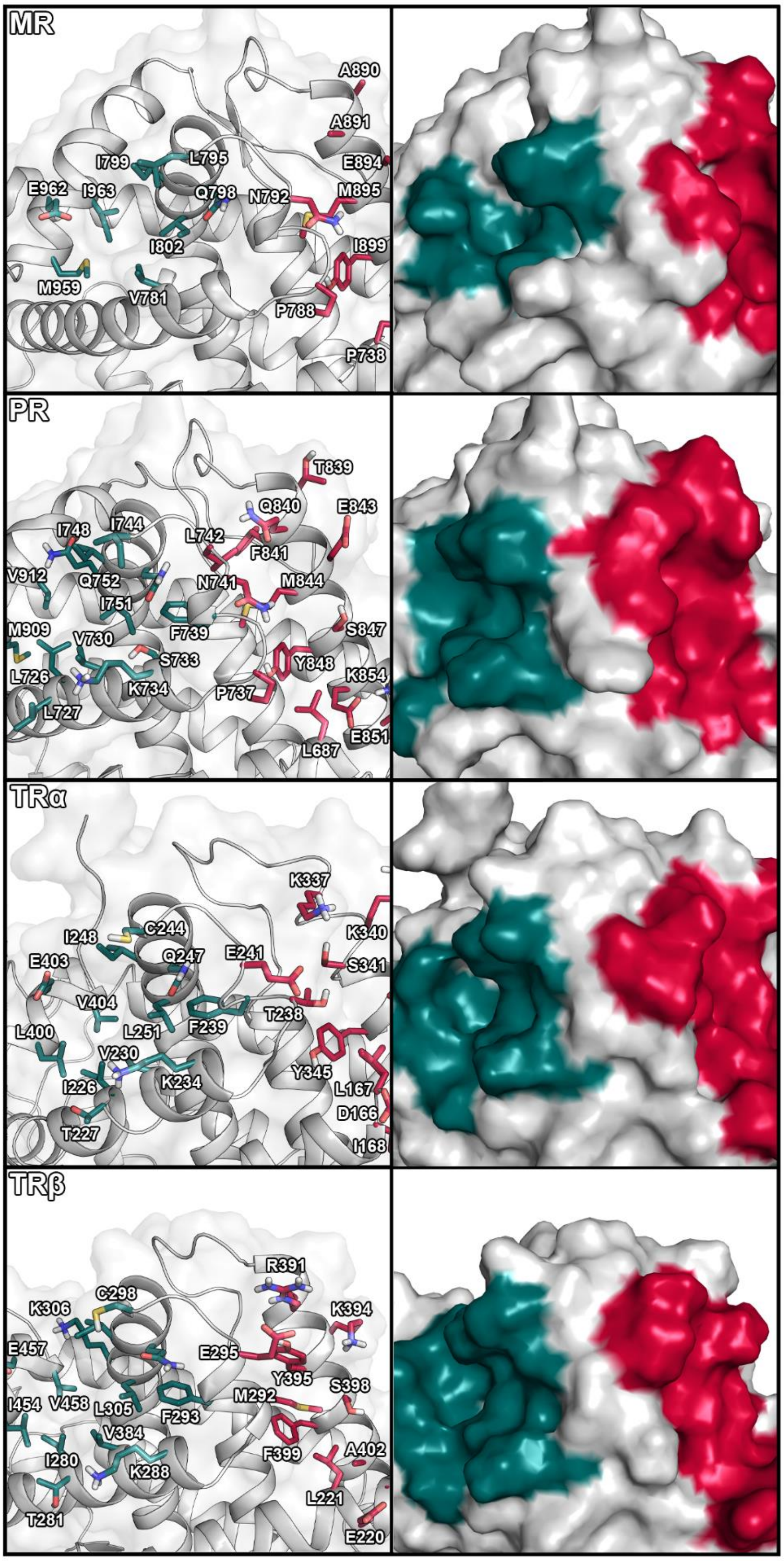

Representation of the AF-2 and BF-3 sites MR, PR, TRα, and TRβ. The AF-2 is shown in pine green, while the BF-3 site was colored red. The surface was colored according to the type of residue (blue, positive charge; red, negative charge; green, non-polar; yellow, cysteine; purple, glycine; light blue, histidine).

Distinct Pharmacophores of the Allosteric Sites

Figure S3. Comparison between cosolvent densities between apo and holo protein for the AF-2 site.

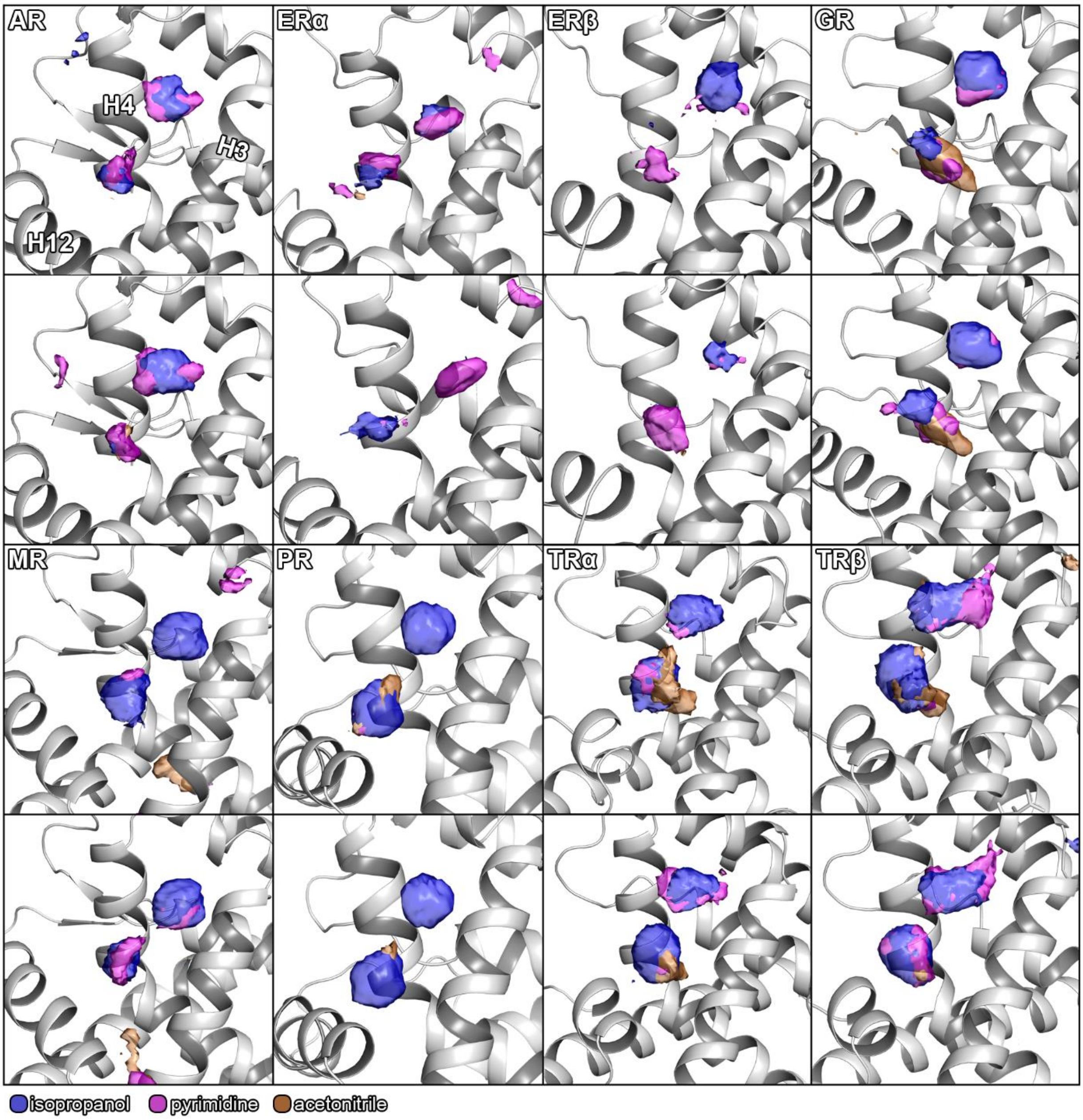

For each receptor, a comparison of the probe densities between holo (upper part) and apo (lower part) structure is shown. The densities are shown at an isovalue of 12. A legend to interpret the colors is given below the figure. The viewpoint was held consistent.

Figure S4. Comparison between cosolvent densities between apo and holo protein for the BF-3 site.

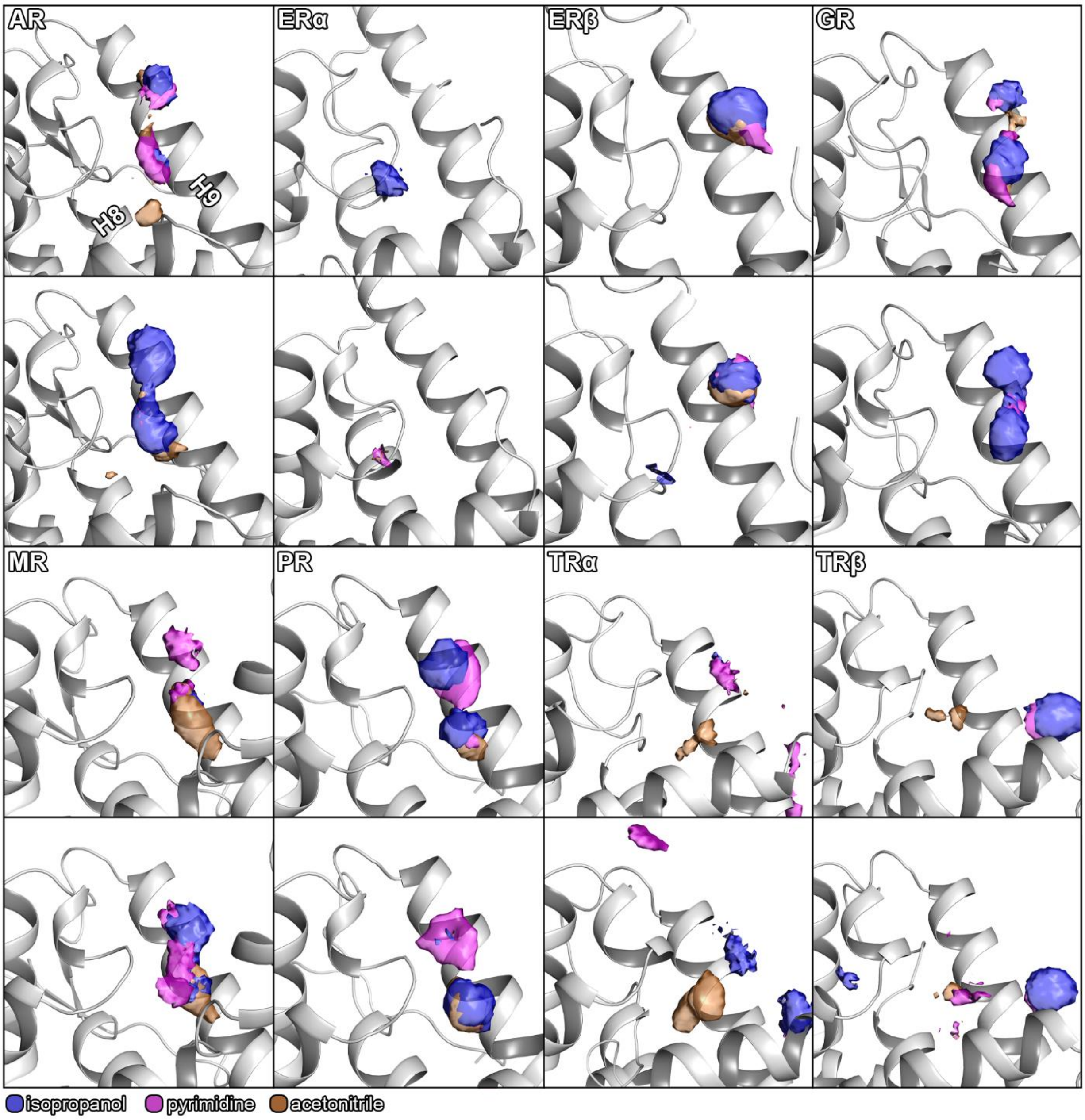

For each receptor, a comparison of the probe densities between holo (upper part) and apo (lower part) structure is shown. The densities are shown at an isovalue of 12. A legend to interpret the colors is given in below the figure. The viewpoint was held consistent.

Table S1. Backbone RMSD of AR cosolvent MD simulations.

Replica	Acetonitrile apo	Isopropanol apo	Pyrimidine apo	Acetonitrile holo	Isopropanol holo	Pyrimidine holo
1	1.30	1.20	1.59	1.14	1.27	1.10
2	1.24	1.33	1.46	1.13	1.07	1.24
3	1.51	1.37	1.66	1.08	1.29	1.23
4	1.44	1.34	1.44	1.16	1.26	1.36
5	1.33	1.50	1.67	1.06	1.40	1.22
6	1.47	1.12	1.56	1.15	1.22	1.27
7	1.55	1.39	1.80	1.08	1.07	1.10
8	1.45	1.14	1.07	1.23	1.25	1.19
9	1.40	1.42	1.37	0.97	1.31	1.13
10	1.46	1.33	1.36	1.49	1.25	1.29

The backbone RMSD (Å) was determined between the input structure of the simulations and the last frame of the respective replica.

Table S2. Backbone RMSD of ERα cosolvent MD simulations.

Replica	Acetonitrile apo	Isopropanol apo	Pyrimidine apo	Acetonitrile holo	Isopropanol holo	Pyrimidine holo
1	1.71	1.37	1.26	1.44	1.36	1.53
2	1.51	2.13	1.37	1.40	1.47	1.20
3	1.24	1.71	1.29	1.81	1.25	1.32
4	1.14	1.24	1.46	1.47	1.43	1.85
5	1.53	1.22	1.45	1.42	1.71	1.55
6	1.54	1.43	1.22	1.44	1.50	1.35
7	1.56	1.77	1.49	1.66	1.24	1.57
8	1.36	1.89	1.37	1.36	1.07	1.81
9	1.38	1.42	1.46	1.04	1.50	1.37
10	1.92	1.34	1.65	1.31	1.19	1.65

The RMSD (Å) was determined between the input structure of the simulations and the last frame of the respective replica.

Table S3. Backbone RMSD of ERβ cosolvent MD simulations.

Replica	Acetonitrile apo	Isopropanol apo	Pyrimidine apo	Acetonitrile holo	Isopropanol holo	Pyrimidine holo
1	2.19	1.61	1.88	1.36	1.48	1.48
2	1.60	1.59	1.29	1.24	1.35	1.67
3	1.90	1.54	1.74	1.81	1.27	1.59
4	1.55	1.74	1.68	1.59	1.28	1.48
5	1.75	1.87	2.01	1.39	1.55	1.45
6	1.88	1.36	1.99	1.45	1.22	1.28
7	1.45	1.79	1.56	1.27	1.81	1.48
8	1.74	1.54	1.43	1.44	1.65	1.40
9	1.44	1.67	1.87	1.64	1.22	1.42
10	1.88	1.51	1.89	1.40	1.19	1.74

The RMSD (Å) was determined between the input structure of the simulations and the last frame of the respective replica.

Table S4. Backbone RMSD of GR cosolvent MD simulations.

Replica	Acetonitrile apo	Isopropanol apo	Pyrimidine apo	Acetonitrile holo	Isopropanol holo	Pyrimidine holo
1	1.30	1.43	1.14	1.30	1.40	1.29
2	1.33	1.36	1.14	1.04	1.28	1.35
3	1.29	1.38	1.07	1.17	1.42	1.36
4	1.27	1.16	1.10	1.22	1.18	1.14
5	1.38	1.29	1.39	1.15	1.26	1.20
6	1.26	1.42	1.21	1.23	1.16	1.19
7	1.24	1.14	1.24	1.15	1.24	1.07
8	1.24	1.51	1.39	1.23	1.08	1.36
9	1.27	1.41	1.35	1.25	1.23	1.25
10	1.36	1.22	1.14	1.33	1.08	1.29

The backbone RMSD (Å) was determined between the input structure of the simulations and the last frame of the respective replica.

Table S5. Backbone RMSD of MR cosolvent MD simulations.

Replica	Acetonitrile apo	Isopropanol apo	Pyrimidine apo	Acetonitrile holo	Isopropanol holo	Pyrimidine holo
1	1.31	1.44	1.86	1.61	1.60	1.46
2	1.56	1.30	1.47	1.69	1.30	1.78
3	1.68	1.76	1.73	1.41	1.51	1.50
4	1.78	1.68	1.88	1.38	1.50	1.37
5	1.67	1.48	1.74	1.48	1.59	1.49
6	1.61	1.69	1.53	1.67	1.58	1.33
7	1.41	1.40	1.84	1.35	1.48	1.78
8	1.39	1.54	1.59	1.49	1.45	1.40
9	1.42	1.43	1.66	1.72	1.59	1.56
10	1.25	1.87	1.70	1.68	1.28	1.36

The backbone RMSD (Å) was determined between the input structure of the simulations and the last frame of the respective replica.

Table S6. Backbone RMSD of PR cosolvent MD simulations.

Replica	Acetonitrile apo	Isopropanol apo	Pyrimidine apo	Acetonitrile holo	Isopropanol holo	Pyrimidine holo
1	1.29	1.10	1.18	1.10	0.87	1.19
2	1.12	1.05	1.04	0.98	0.81	1.28
3	0.98	1.26	1.06	1.07	1.04	0.91
4	1.03	0.97	1.21	1.00	1.06	0.90
5	1.07	0.89	1.17	1.17	1.02	1.09
6	1.04	1.41	1.02	1.12	1.29	1.03
7	1.14	1.14	1.23	1.05	1.40	1.30
8	1.07	1.23	1.06	1.05	1.05	1.03
9	1.18	0.91	1.26	0.98	1.02	1.27
10	1.16	1.13	1.14	1.05	1.07	1.12

The backbone RMSD (Å) was determined between the input structure of the simulations and the last frame of the respective replica.

Table S7. Backbone RMSD of TRα cosolvent MD simulations.

Replica	Acetonitrile apo	Isopropanol apo	Pyrimidine apo	Acetonitrile holo	Isopropanol holo	Pyrimidine holo
1	2.15	1.82	1.96	1.84	1.67	2.41
2	1.85	1.45	1.75	1.97	2.02	1.24
3	1.86	1.30	1.64	2.03	2.28	2.01
4	1.64	2.12	1.90	1.60	1.32	1.51
5	1.94	2.57	1.56	1.69	2.35	2.23
6	2.37	1.91	1.86	2.44	1.69	2.11
7	2.03	1.76	2.25	1.97	1.61	1.82
8	1.74	2.01	2.21	1.82	1.93	1.72
9	1.71	1.73	1.93	1.91	2.32	1.82
10	2.03	1.85	1.63	1.88	1.92	2.08

The backbone RMSD (Å) was determined between the input structure of the simulations and the last frame of the respective replica.

Table S8. Backbone RMSD of TRβ cosolvent MD simulations.

Replica	Acetonitrile apo	Isopropanol apo	Pyrimidine apo	Acetonitrile holo	Isopropanol holo	Pyrimidine holo
1	1.51	1.49	1.38	1.51	1.85	1.95
2	1.42	1.77	1.75	1.55	1.40	1.64
3	1.56	1.72	1.51	1.54	1.55	1.69
4	1.64	1.24	1.32	1.51	1.51	1.68
5	1.87	1.61	1.73	1.55	1.69	1.91
6	1.58	1.90	1.50	1.71	1.70	1.61
7	1.48	1.65	1.32	1.55	1.31	1.74
8	1.63	1.62	1.43	1.56	1.65	1.59
9	1.48	1.40	1.73	1.65	1.66	1.60
10	1.63	1.74	1.73	1.59	1.51	1.31

The backbone RMSD (Å) was determined between the input structure of the simulations and the last frame of the respective replica.

Conformational Change

Figure S5. Conformational change at AF-2 and BF-3 crystal structures.

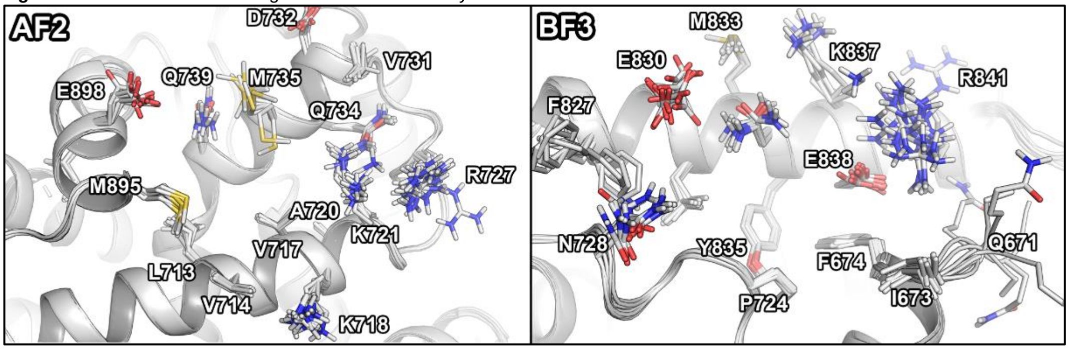

Superposition of holo crystal structures of the allosteric site (PDB IDs: 2PIP, 2PIV, 2YHD, 2YLO, 2YLP, 2PIT, 2PIU, 2PIO, 2PKL, 2YLQ, 2PIW, 4HLW)

Figure S6. Conformational change at AF-2 and BF-3 determined by RMSD.

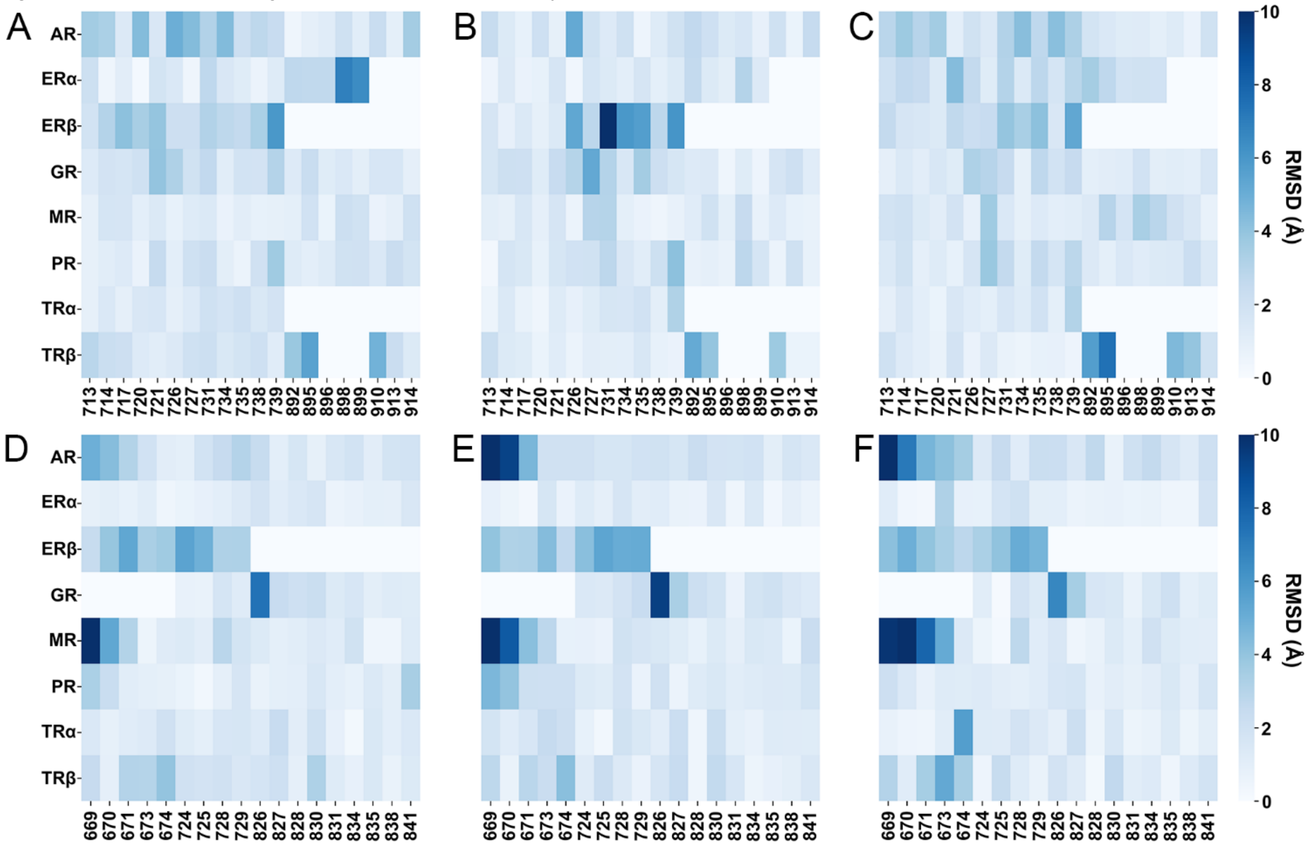

The RMSD between the representative structures of cosolvent and pure water simulations is shown for (A) AF-2 site in acetonitrile, (B) AF-2 in isopropanol, (B) AF-2 in pyrimidine, (D) BF-3 in acetonitrile, (E) BF-3 in isopropanol, and (F) BF-3 in pyrimidine.

Figure S7. RMSD of simulations in pure water.

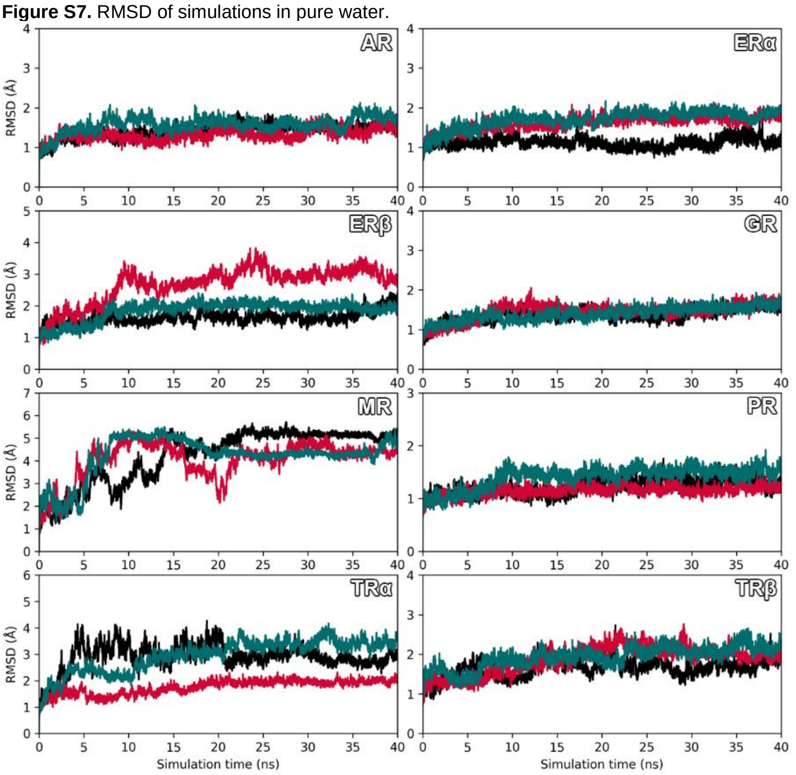

The backbone RMSD of simulations in pure water (performed in triplicates) is presented for each receptor.

Hydration Sites of the Allosteric Sites

Figure S8. Hydration sites determined from crystal structure analysis.

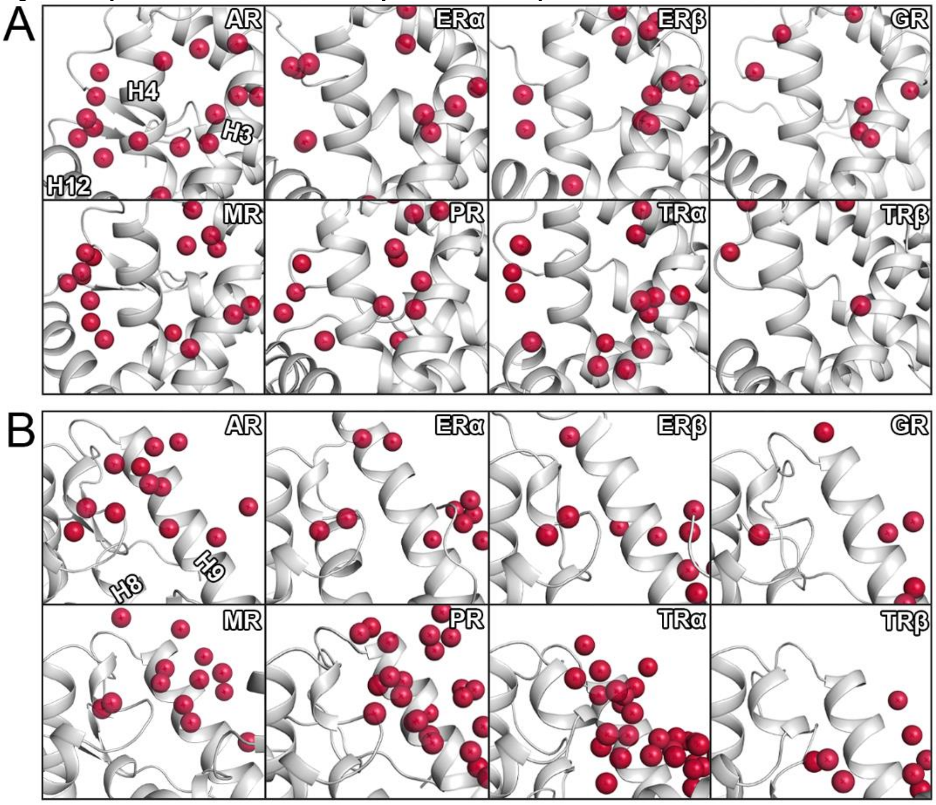

The hydration sites determined to be conserved in the hydration site analysis based on crystal structures. While (A) highlights the AF-2 site the (B) panel presents the BF-3 site.

Figure S9. Hydration sites determined from crystal structure analysis.

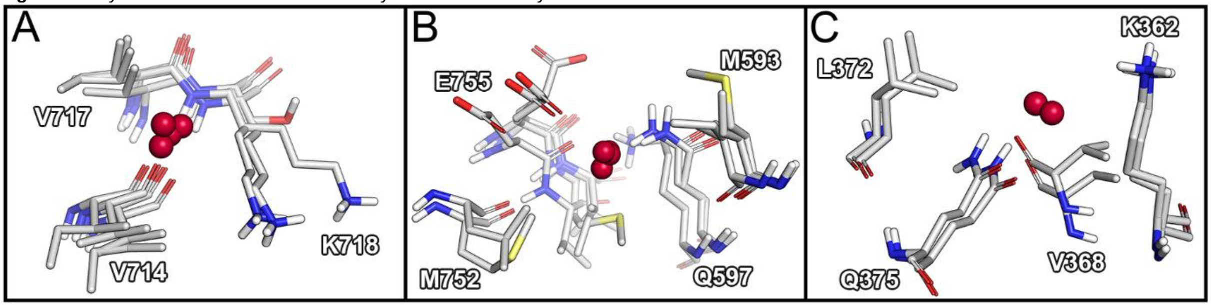

(A) Hydration site conserved among AR, ERβ, GR and MR. (B) Hydration site conserved among ERβ, GR, PR, TRβ. (C) Hydration site conserved among ERs. The nomenclature for the shown residues was selected based on (A) AR, (B) GR, and (C) ERα.

Figure S10. RMSD analysis of WATsite simulations.

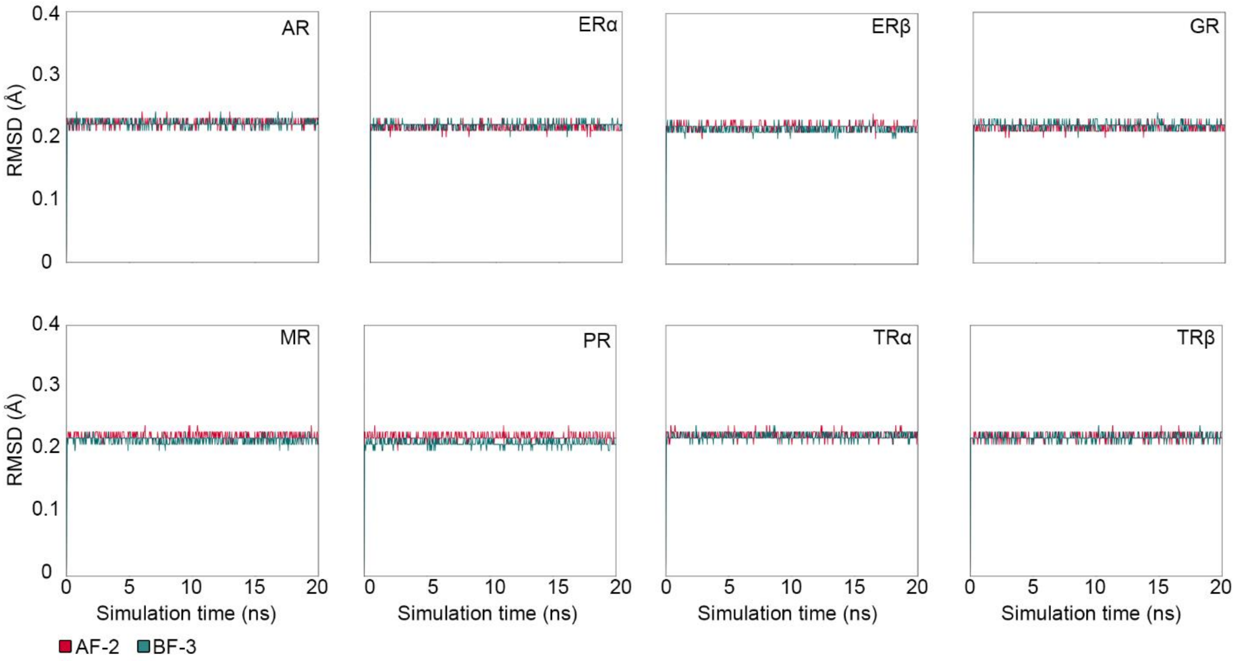

The backbone RMSD of WATsite simulations is presented for each receptor. Since a separate simulation was performed for each site, different colors were used to indicate the respective simulation.

Table S9. Results from the hydration site analysis using WATsite for the AR.

Site	Hydration site	Enthalpy ΔH (kcal/mol)	Entropy -T*ΔS (kcal/mol)
AF-2	1	-0.494	1.242
	2	-1.776	2.283
	3	-0.804	1.546
	4	3.317	1.039
	5	0.396	1.891
	6	-1.051	1.247
	7	0.697	1.242
	8	1.938	1.232
	9	3.129	1.951
	10	0.762	1.037
	11	0.397	1.134
	12	2.685	1.514
	13	0.876	1.022
	14	-0.157	1.138
	15	-0.306	1.338
	16	1.105	1.280
	17	-1.167	1.799
	18	1.060	1.985
	19	0.663	1.237
	20	2.341	2.256
	21	4.476	1.851
BF-3	1	-0.034	1.078
	2	0.027	1.205
	3	4.036	1.393
	4	0.442	1.251
	5	-3.028	1.323
	6	1.624	2.949
	7	-0.946	1.510
	8	0.378	0.826
	9	-2.587	1.394
	10	-3.142	1.758
	11	-0.358	2.578
	12	-1.936	1.841
	13	-2.465	1.193
	14	1.714	0.810
	15	-2.561	1.347
	16	-0.541	2.307
	17	2.653	1.143
	18	-2.133	1.276
	19	0.986	1.018
	20	-2.732	1.921
	21	-0.579	1.131
	22	-0.993	1.486
	23	-2.741	1.400
	24	-2.128	2.719
	25	0.412	1.001
	26	0.797	1.657
	27	-0.446	1.274
	28	-0.109	1.390
	29	-0.617	1.506
	30	-0.832	1.838
	31	-1.135	1.839
	32	0.256	1.996

Together with supplied PDB files, the contributions for displaced water molecules can be determined.

Table S10. Results from the hydration site analysis using WATsite for the ERα.

Site	Hydration site	Enthalpy ΔH (kcal/mol)	Entropy -T*ΔS (kcal/mol)
AF-2	1	1.686	1.337
	2	-0.022	1.843
	3	-1.204	2.276
	4	-0.949	1.092
	5	1.875	1.729
	6	-0.098	1.159
	7	-0.687	1.509
	8	1.390	1.083
	9	0.784	1.085
	10	0.157	2.056
	11	-0.061	1.277
	12	2.390	1.538
	13	1.456	1.500
	14	-0.260	1.812
	15	6.644	3.013
	16	-0.039	1.398
BF-3	1	5.677	1.466
	2	-1.973	1.978
	3	0.662	1.664
	4	1.298	1.559
	5	-4.308	2.762
	6	1.416	1.238
	7	-3.094	2.316
	8	0.428	0.876
	9	-1.836	1.438
	10	-1.805	1.362
	11	1.097	2.862
	12	-0.945	2.275
	13	-2.299	2.269
	14	-2.839	3.310
	15	-3.174	1.960
	16	-1.077	1.421
	17	-0.528	1.391
	18	-1.890	1.626
	19	0.536	1.206
	20	-3.071	1.938
	21	0.640	1.240
	22	9.328	2.345
	23	-2.368	2.278

Together with supplied PDB files, the contributions for displaced water molecules can be determined.

Table S11. Results from the hydration site analysis using WATsite for the ERβ.

Site	Hydration site	Enthalpy ΔH (kcal/mol)	Entropy -T*ΔS (kcal/mol)
AF-2	1	-2.528	1.464
	2	0.318	1.260
	3	1.129	1.437
	4	-0.920	1.619
	5	1.322	2.024
	6	-0.514	1.446
	7	-1.732	1.462
	8	0.288	1.003
	9	-0.073	1.051
	10	-0.694	1.898
	11	-1.062	0.897
	12	-2.424	1.266
	13	-0.589	1.416
	14	3.443	1.117
	15	1.127	1.003
	16	3.085	3.519
	17	0.101	1.432
	18	0.103	0.995
	19	0.350	1.618
	20	0.650	1.423
	21	0.827	1.379
	22	-0.044	1.358
	23	-3.236	2.227
	24	-0.219	1.386
	25	-0.984	2.146
BF-3	1	0.115	1.357
	2	-1.174	1.054
	3	-0.095	1.834
	4	0.166	1.373
	5	-3.478	1.714
	6	1.053	1.285
	7	3.743	1.162
	8	0.872	1.109
	9	1.778	2.816
	10	-3.028	1.879
	11	-3.195	2.644
	12	0.454	1.252
	13	-1.191	1.018
	14	-0.243	1.976
	15	0.480	0.666
	16	0.195	1.209
	17	-0.939	2.012
	18	0.823	1.018
	19	-3.589	2.081
	20	0.613	1.636
	21	-0.801	1.331
	22	-0.057	2.372
	23	-0.722	1.071
	24	1.203	1.869
	25	-0.719	1.355
	26	2.975	1.397
	27	-0.567	1.330
	28	-0.931	1.477
	29	-4.742	3.268
	30	-0.823	1.087
	31	2.564	1.403
	32	0.435	1.403
	33	-0.368	1.414

Together with supplied PDB files, the contributions for displaced water molecules can be determined.

Table S12. Results from the hydration site analysis using WATsite for the GR.

Site	Hydration site	Enthalpy ΔH (kcal/mol)	Entropy -T*ΔS (kcal/mol)
AF-2	1	-1.450	1.888
	2	0.020	1.125
	3	-3.132	2.182
	4	0.835	1.778
	5	-1.745	1.478
	6	-1.440	2.450
	7	-0.312	1.337
	8	-0.371	1.123
	9	2.096	2.127
	10	3.194	2.106
	11	-0.748	1.273
	12	4.567	1.494
	13	0.982	1.704
	14	0.454	0.994
	15	0.672	1.501
	16	0.742	1.443
	17	-0.151	0.982
	18	-0.179	1.510
	19	-0.635	1.819
	20	1.841	1.301
	21	2.310	1.707
	22	3.269	1.373
	23	-2.419	1.809
	24	2.854	1.584
	25	1.190	1.660
	26	-0.165	1.510
	27	1.481	1.668
	28	1.431	1.546
BF-3	1	-1.712	2.325
	2	-2.350	1.518
	3	3.088	1.496
	4	-2.556	1.360
	5	-3.629	1.710
	6	-0.872	0.893
	7	-2.010	1.060
	8	1.369	0.979
	9	-2.627	1.800
	10	-2.046	1.477
	11	-0.318	2.084
	12	0.397	1.272
	13	-1.834	1.544
	14	0.199	1.060
	15	-3.091	1.143
	16	0.154	1.144
	17	0.013	1.204
	18	1.780	1.194
	19	0.093	1.453
	20	-1.147	1.779
	21	1.433	1.252

Together with supplied PDB files, the contributions for displaced water molecules can be determined.

Table S13. Results from the hydration site analysis using WATsite for the MR.

Site	Hydration site	Enthalpy ΔH (kcal/mol)	Entropy -T*ΔS (kcal/mol)
AF-2	1	-1.166	1.477
	2	-0.375	1.303
	3	-0.090	1.126
	4	-2.637	1.218
	5	0.793	1.575
	6	0.478	2.968
	7	-0.069	0.927
	8	-1.421	1.326
	9	0.037	1.172
	10	-1.355	1.187
	11	-0.162	1.219
	12	-2.835	1.126
	13	1.587	1.051
	14	-0.757	1.146
	15	-0.114	1.189
	16	-1.896	3.226
	17	-0.150	1.166
	18	-0.687	1.048
	19	2.746	2.030
	20	3.404	1.203
	21	3.343	1.942
	22	0.373	2.113
	23	-3.081	1.464
	24	0.787	2.502
	25	0.249	1.435
	26	0.233	1.540
BF-3	1	0.423	1.414
	2	-0.074	1.423
	3	-0.038	1.282
	4	-2.404	2.225
	5	0.107	1.129
	6	2.132	1.807
	7	0.590	1.446
	8	-1.245	1.665
	9	-0.117	1.253
	10	-0.234	1.883
	11	-1.503	2.347
	12	-1.689	2.307
	13	-2.423	1.259
	14	-2.835	1.377
	15	-2.114	2.422
	16	0.308	0.999
	17	-2.694	1.385
	18	-0.889	1.221
	19	-1.028	0.926
	20	-0.138	1.740
	21	0.422	1.058
	22	2.315	1.752
	23	-2.266	1.838
	24	-1.022	1.392
	25	1.550	1.128
	26	0.220	1.879
	27	-1.076	1.350

Together with supplied PDB files, the contributions for displaced water molecules can be determined.

Table S14. Results from the hydration site analysis using WATsite for the PR.

Site	Hydration site	Enthalpy ΔH (kcal/mol)	Entropy -T*ΔS (kcal/mol)
AF-2	1	1.899	1.136
	2	1.525	1.982
	3	0.082	1.417
	4	0.493	1.025
	5	-0.078	1.647
	6	-0.241	1.120
	7	-0.589	1.269
	8	2.706	1.177
	9	-0.851	0.998
	10	0.256	2.410
	11	2.686	1.259
	12	-0.800	1.389
	13	-0.218	1.239
	14	0.039	1.163
	15	1.101	1.005
	16	1.029	1.212
	17	-0.487	1.047
	18	1.156	2.287
	19	3.025	1.309
	20	2.006	1.195
	21	0.183	0.881
	22	5.526	1.842
	23	0.572	1.643
	24	0.346	1.279
	25	0.490	1.375
	26	-0.080	1.364
	27	-0.750	1.391
	28	0.912	2.620
BF-3	1	-1.176	2.282
	2	-0.630	1.493
	3	-0.173	1.252
	4	-2.577	1.573
	5	-2.831	1.361
	6	-2.604	1.564
	7	-1.280	1.581
	8	-1.544	2.422
	9	-0.169	1.246
	10	-1.239	1.657
	11	-2.005	2.672
	12	-0.481	1.416
	13	2.363	1.439
	14	-1.456	1.780
	15	-2.933	4.050
	16	-1.956	1.513
	17	0.748	0.992
	18	0.497	0.954
	19	-0.062	1.049
	20	-1.115	1.695
	21	0.474	1.569
	22	-3.270	1.340
	23	0.711	1.106
	24	0.355	1.318
	25	0.800	1.119
	26	-0.977	1.029
	27	-0.462	1.466
	28	-0.014	1.321
	29	0.298	1.286
	30	-2.060	2.165
	31	-0.179	3.041

Together with supplied PDB files, the contributions for displaced water molecules can be determined.

Table S15. Results from the hydration site analysis using WATsite for the TRα.

Site	Hydration site	Enthalpy ΔH (kcal/mol)	Entropy -T*ΔS (kcal/mol)
AF-2	1	-2.281	1.745
	2	-0.881	1.467
	3	-0.209	1.044
	4	0.073	1.066
	5	1.593	1.456
	6	-0.263	0.945
	7	-0.385	1.312
	8	-1.648	2.518
	9	1.283	2.482
	10	-1.949	1.531
	11	1.170	1.084
	12	0.612	2.079
	13	0.045	1.495
	14	-0.874	0.987
	15	0.317	1.213
	16	1.138	1.210
	17	-3.192	1.621
	18	1.022	1.079
	19	-1.673	2.313
	20	-2.714	1.572
	21	-2.047	1.568
	22	2.491	1.467
	23	-0.582	1.076
	24	-0.509	1.492
	25	-0.282	1.453
	26	-0.482	1.439
BF-3	1	-1.910	1.147
	2	0.699	0.996
	3	-0.006	1.014
	4	0.149	2.436
	5	-1.087	0.928
	6	-0.719	1.004
	7	-2.322	2.107
	8	-0.054	1.250
	9	-2.651	1.857
	10	-0.151	0.993
	11	0.695	0.940
	12	0.144	4.222
	13	1.439	2.201
	14	-1.717	1.053
	15	0.631	1.180
	16	-0.594	1.340
	17	-0.126	1.164
	18	0.259	1.302
	19	-4.333	2.419
	20	-3.912	4.797
	21	-1.692	1.279
	22	-1.958	1.322
	23	5.827	3.775
	24	0.815	1.770
	25	-1.911	2.939
	26	1.430	4.353
	27	0.016	1.486
	28	3.330	3.112

Together with supplied PDB files, the contributions for displaced water molecules can be determined.

Table S16. Results from the hydration site analysis using WATsite for the TRβ.

Site	Hydration site	Enthalpy ΔH (kcal/mol)	Entropy -T*ΔS (kcal/mol)
AF-2	1	-0.950	2.188
	2	2.440	2.059
	3	0.776	0.772
	4	1.657	1.084
	5	-0.851	1.706
	6	0.651	1.147
	7	1.646	1.030
	8	2.202	1.809
	9	4.439	2.164
	10	-0.630	1.677
	11	0.124	1.098
	12	2.192	1.177
	13	0.712	1.333
	14	-0.165	1.103
	15	0.053	1.359
	16	0.271	1.174
	17	-0.313	1.656
	18	-2.557	1.708
	19	-0.735	2.331
	20	-2.922	1.945
BF-3	1	-0.489	1.175
	2	2.328	2.076
	3	-0.399	1.179
	4	2.350	1.311
	5	0.439	1.970
	6	-1.945	1.732
	7	-0.701	1.558
	8	-1.152	1.157
	9	-3.593	2.143
	10	-0.903	2.736
	11	-0.823	1.223
	12	-6.124	3.778
	13	-0.618	1.306
	14	-2.101	1.378
	15	1.404	2.224
	16	-1.042	2.569
	17	-0.930	1.257
	18	0.597	1.198
	19	0.178	1.290
	20	5.749	4.000
	21	-1.485	1.554
	22	-4.054	2.392
	23	0.961	0.967
	24	0.264	1.209
	25	-2.831	1.930
	26	0.177	1.378
	27	-0.084	1.265
	28	2.278	3.272
	29	-0.208	1.240
	30	0.152	1.578
	31	-0.369	1.134
	32	-0.261	2.330
	33	-1.878	4.147
	34	0.328	1.493
	35	2.981	1.828
	36	-0.110	1.351
	37	-0.100	1.447

Together with supplied PDB files, the contributions for displaced water molecules can be determined.

Molecular Docking

Figure S11. Poses obtained from redocking known crystallographic ligands: Glide SP for the AF-2 site

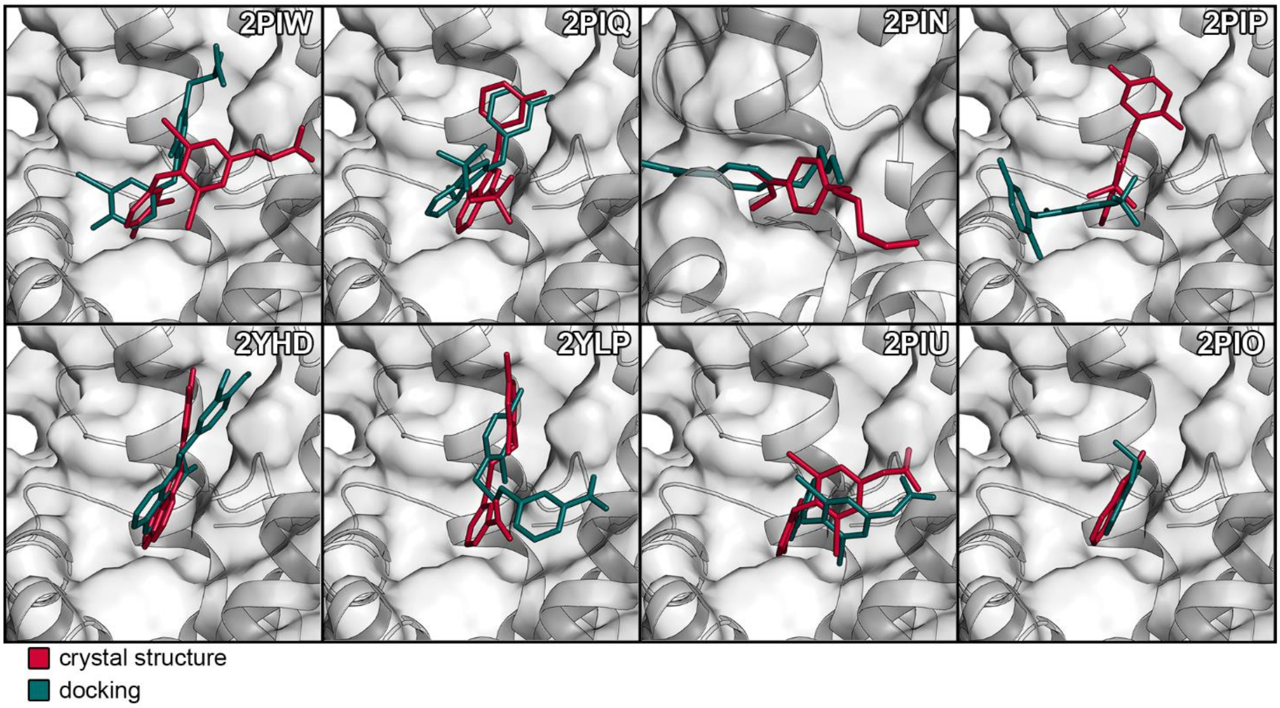

Figure S12. Poses obtained from redocking known crystallographic ligands: Glide SP for the BF-3 site.

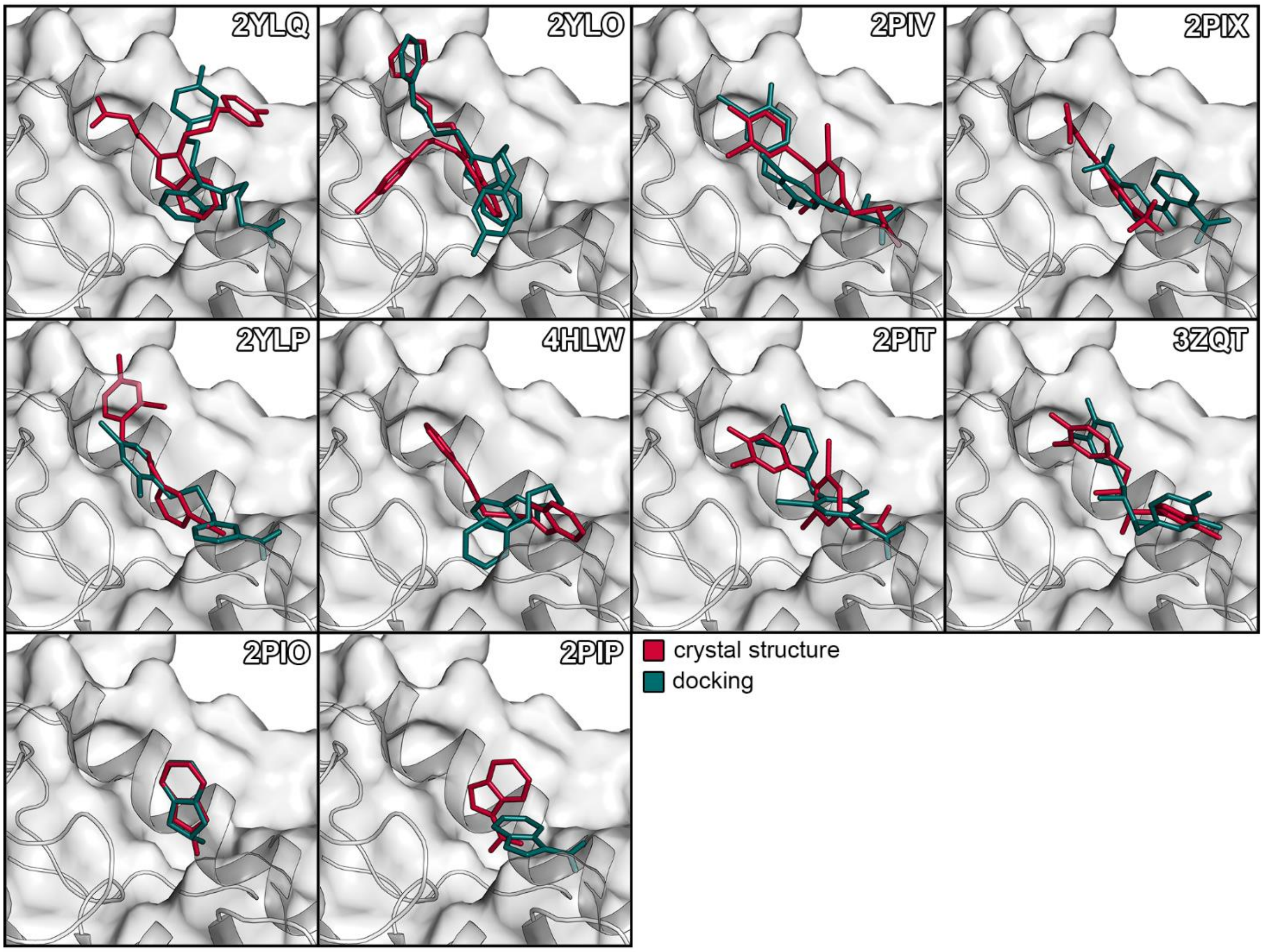

Figure S13. Poses obtained from redocking known crystallographic ligands: Glide XP for the AF-2 site.

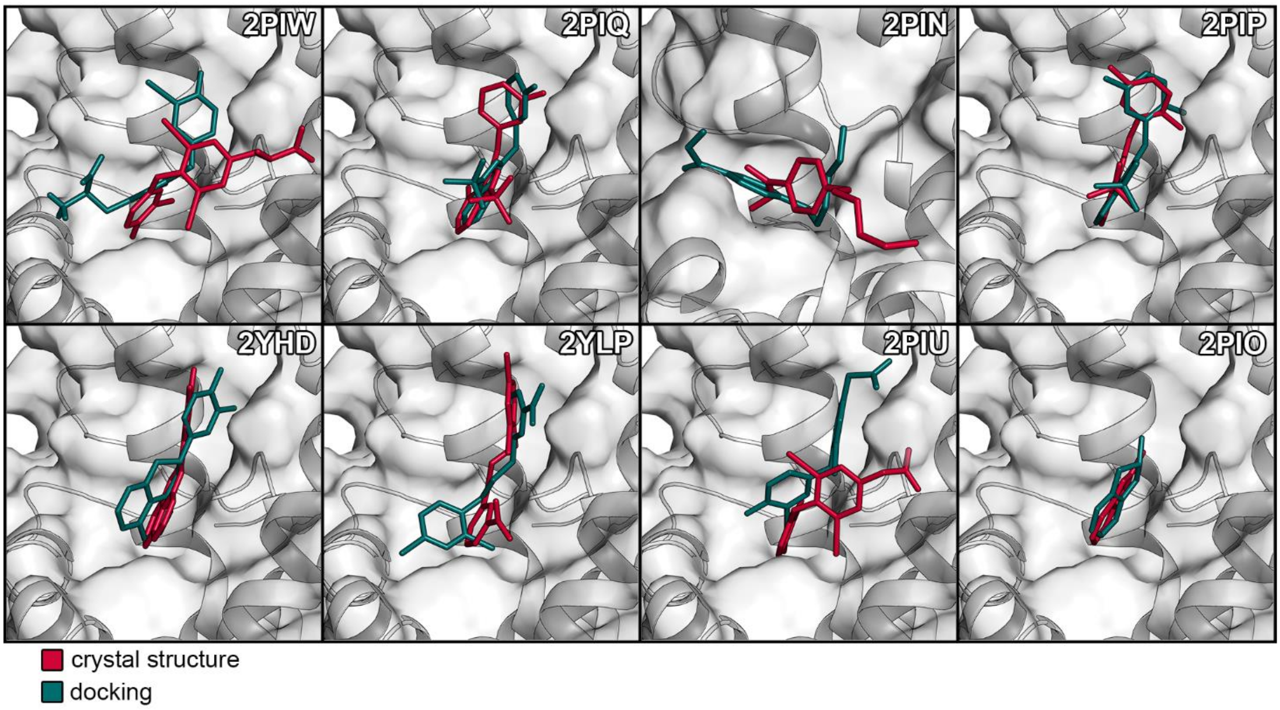

Figure S14. Poses obtained from redocking known crystallographic ligands: Glide XP for the AF-2 site.

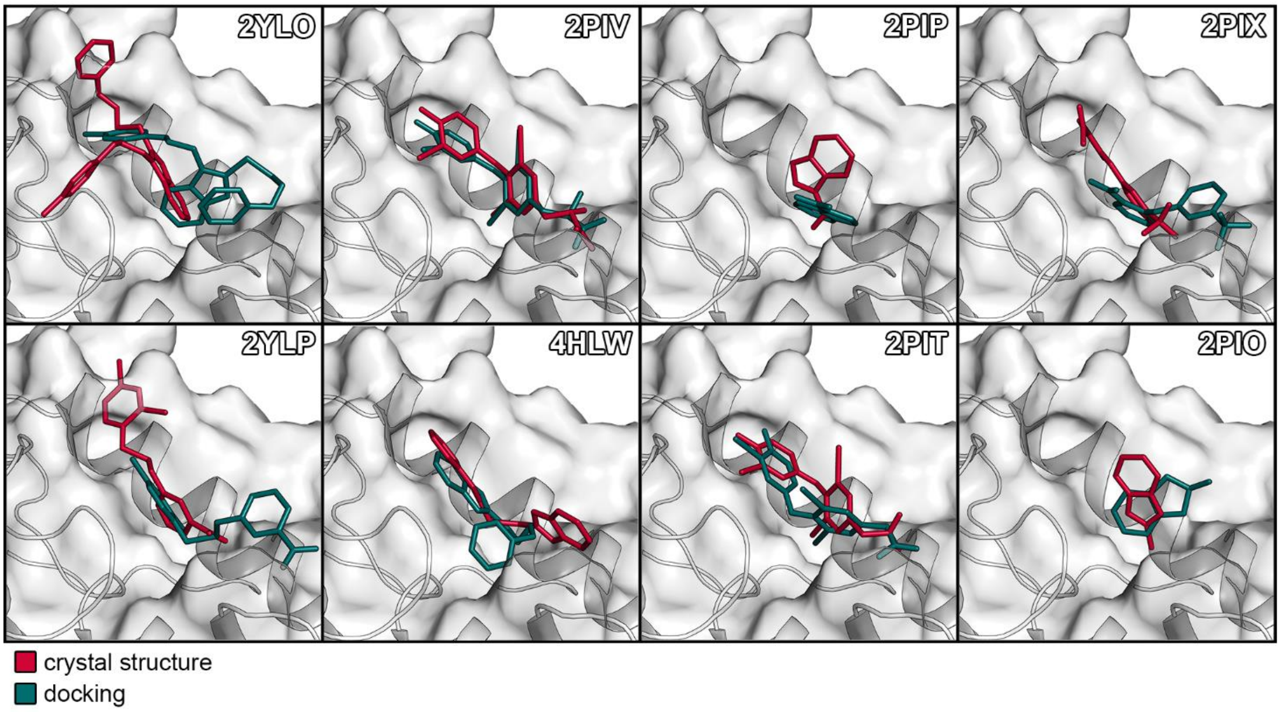

Table S17. RMSD obtained from redocking known crystallographic ligands.

PDB ID	Site	RMSD SP[a] (Å)	RMSD XP[b] (Å)
2PIQ	AF-2	2.11	1.19
2YHD	AF-2	1.47	1.96
2PIW	AF-2	4.90	7.92
2PIP	AF-2	7.10	0.84
2YLP	AF-2	4.67	7.24
2PIU	AF-2	2.24	4.28
2PIO	AF-2	1.67	0.92
2YLQ	BF-3	6.76	n/a[c]
2PIX	BF-3	7.46	5.06
2YLP	BF-3	5.22	7.10
2PIP	BF-3	5.15	4.75
4HLW	BF-3	4.78	7.00
2PIO	BF-3	1.25	3.56
2PIV	BF-3	2.11	1.07
2YLO	BF-3	3.83	8.22
2PIT	BF-3	2.11	1.90
3ZQT	BF-3	1.76	n/a[c]
2PIN	AF-2	4.83	4.91

[a]Results obtained using SP docking protocol.
[b]Results obtained using XP docking protocol.
[c]No pose was obtained by the applied protocol and specifications.

Figure S15. Crystal mates around the BF-3 site.

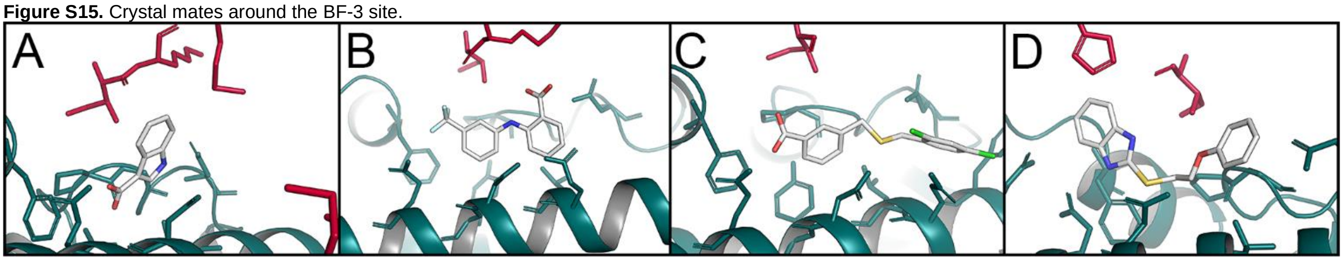

Crystal mates in the 4 A radius of cocrystallized ligands at the BF-3. Nearby mates were colored red.

Table S18. Results from docking the DUD-E dataset against known actives.

	AR AF-2	AR BF-3	ERα AF-2	ERβ AF-2	TRα AF-2	TRβ AF-2	GR AF-2
Actives	44	87	65	3	28	99	8
Decoys	2650	4350	4957	200	1450	5350	450
ROC AUC	0.75	0.76	0.71	0.85	0.45	0.55	0.87

Figure S16. Score distributions for the ERα.

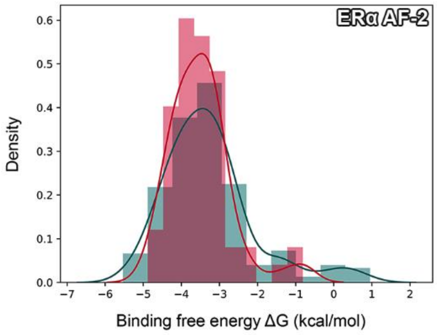

Figure S17. Score distributions determined by the Glide XP docking protocol.

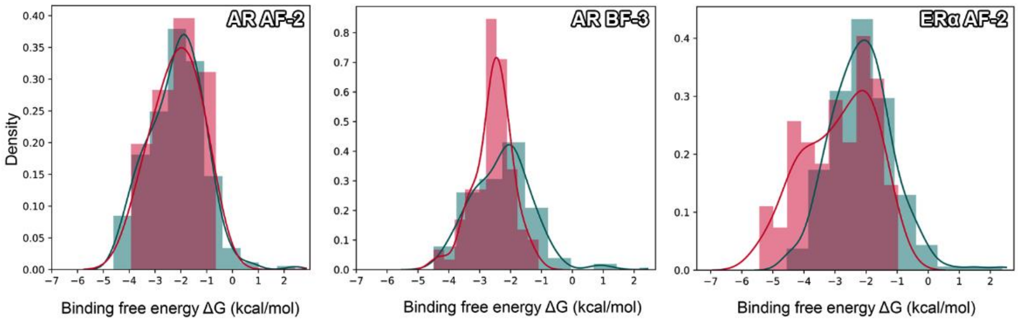

Figure S18. VPC16606 in docked to various NRs.

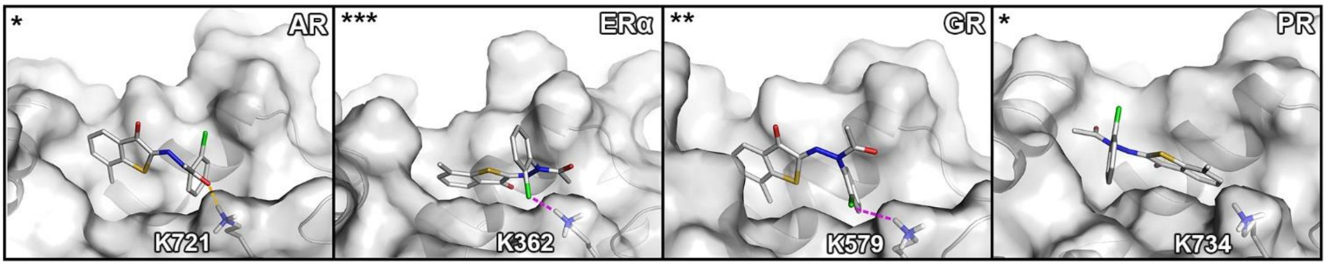

The ERα AF-2 inhibitor VPC166606 was docked into various nuclear receptors. The inhibitory activity measured for each receptor is indicated at the top left by asterisks.

Supporting Materials and Methods

Sequence Alignment and Analysis

Figure S19. Sequence alignment of all NRs assessed in this study.

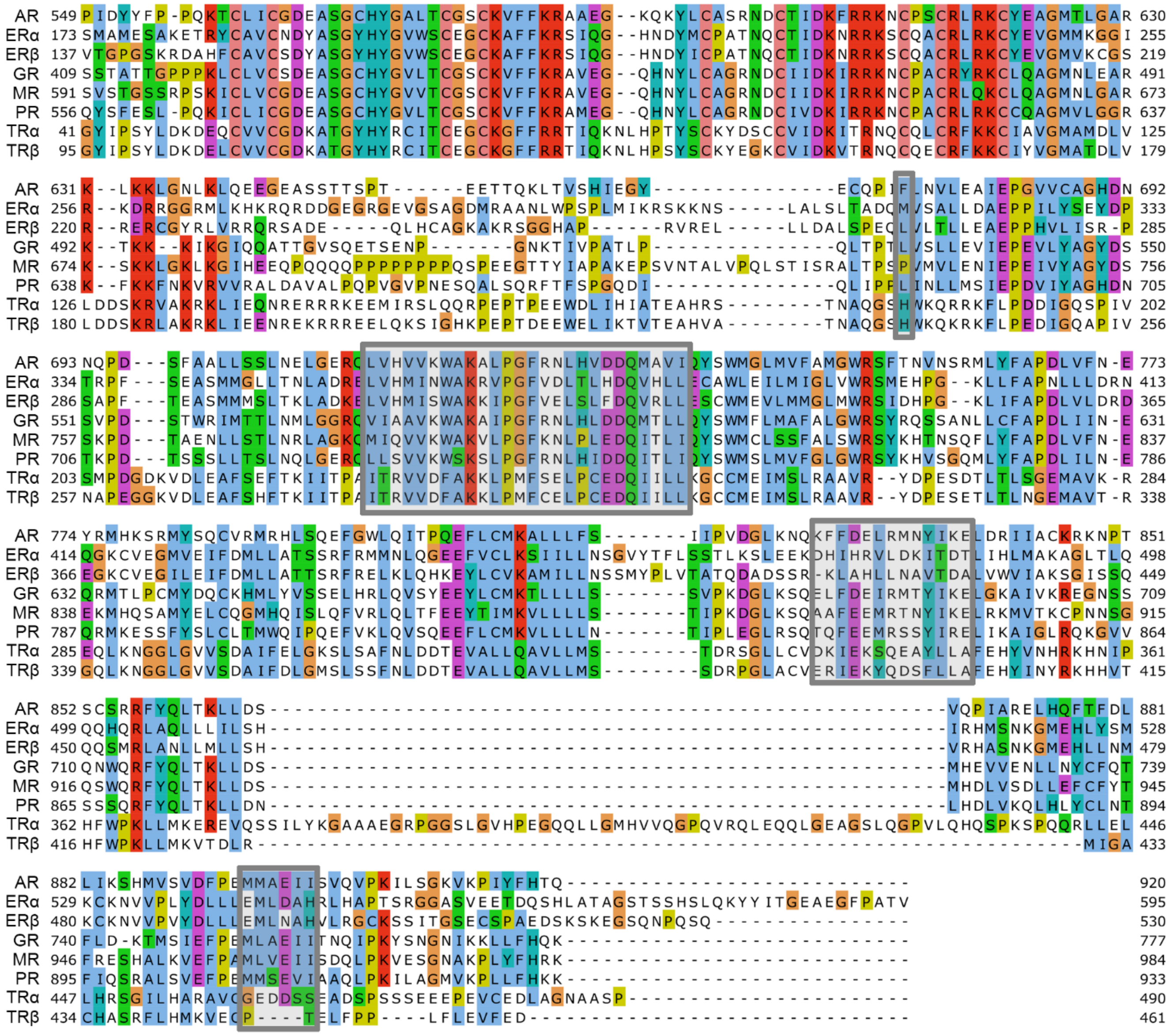

Sequence alignment of the NRs considered in this study. Residues of the allosteric sites were indicated with gray boxes.

Table S19. Amino acid groups for sequence analysis.

Group	Amino acids
1	A, I, L, M, F, W, V, C
2	N, Q, S, T
3	E, D
4	K, R
5	H, Y
6	P
7	G

The amino acids groups used to determine the degree of conservation according to the ClustalW scheme are shown. The residues are given in single-letter code.

Ligand Preparation

Table S20. Structures prepared for molecular docking.

#	SMILES code[a]	Site	Reference[b]
1	[O-]C(=O)c1cc(ccc1)CSCc2c(Cl)cc(Cl)cc2	AR AF-2	[1]
2	CCOc1c(O)ccc(c1)C(Nc2ccc3)Nc4cccc3c24	AR AF-2	[2]
3	c12c3cccc1NC(Nc2ccc3)c4ccc(O)cc4	AR AF-2	[2]
4	COc1c(O)ccc(c1)C(Nc2ccc3)Nc4cccc3c24	AR AF-2	[2]
5	COc1c(O)cc(cc1)C(Nc2ccc3)Nc4cccc3c24	AR AF-2	[2]
6	c12c3cccc1NC(Nc2ccc3)c(c4)ccc(O)c4O	AR AF-2	[2]
7	[O-]C(=O)c1c(O)cc(cc1)N\N=C\2C(=O)N(N=C2C)c3ccccc3	AR AF-2	[2]
8	Cc(c1)[nH]c(c12)cccc2	AR AF-2	[3]
9	Nc1ncnc(c12)n(C(C)(C)C)nc2Cc3c(C)ccc(C)c3	AR AF-2	[3]
10	c1cccc(c1C([O-])=O)Nc(c(Cl)c2C)c(Cl)cc2	AR AF-2	[3]
11	Nc1ncnc(c12)n(C(C)(C)C)nc2Cc3cc(O)ccc3	AR AF-2	[3]
12	[O-]C(=O)[C@@H]([NH3+])Cc(cc1I)cc(I)c1Oc(cc2)cc(I)c2O	AR AF-2	[3]
13	c1cccc(c1C([O-])=O)Nc(c2C)cccc2Cl	AR AF-2	[3]
14	[O-]C(=O)Cc(cc1I)cc(I)c1Oc(cc2)cc(I)c2O	AR AF-2	[3]
15	c1ccccc1CNc(n2)nc(NCC(C)C)cc2CCc3ccccc3	AR AF-2	[5]
16	CC(C)CCc1cc(NCC(C)C)nc(n1)NCC(C)C	AR AF-2	[5]
17	CC(C)CNC(=N1)NC(CCC(C)C)C=C1NCc2ccccc2	AR AF-2	[5]
18	c1ccccc1CCC(NC(=N2)NCC(C)C)C=C2NCc3ccccc3	AR AF-2	[5]
19	CC(C)CNc(n1)nc(NCC(C)C)cc1CCc2cccc(c23)cccc3	AR AF-2	[5]
20	c1ccccc1CCc2cc(NCC(C)C)nc(n2)NCc3cccc(c34)cccc4	AR AF-2	[5]
21	c1ccccc1CNC2=CC(NC(=N2)NCC(C)C)CCc3cccc(c34)cccc4	AR AF-2	[5]
22	c1ccccc1CNc(n2)nc(NCC(C)C)cc2CCc3cccc(c34)cccc4	AR AF-2	[5]
23	c1cccc(c12)cccc2CNc(n3)nc(NCC(C)C)cc3CCc4cccc(c45)cccc5	AR AF-2	[5]
24	c1cccc(c12)cccc2CNC(=N3)NC(CCC(C)C)C=C3NCc4cccc(c45)cccc5	AR AF-2	[5]
25	c1ccccc1CNc2cc(NCc3ccccc3)nc(n2)NCc4ccccc4	AR AF-2	[5]
26	c1cccc(c12)cccc2CCC(C=C3NCc4ccccc4)NC(=N3)NCc5ccccc5	AR AF-2	[5]
27	c1cccc(c12)cccc2CCC(NC(=N3)NCc4ccccc4)C=C3NCc5cccc(c56)cccc6	AR AF-2	[5]
28	c1cccc(c12)cc(O)c(c2)C(=O)N\N=C\c(c3)c(O)cc(c34)cccc4	AR AF-2	[4]
29	c1cccc(c12)cc(O)c(c2)C(=O)N\N=C\c3c(O)ccc(Br)c3	AR AF-2	[4]
30	COc(c1)c(O)c(OC)cc1/C=N/NC(=O)c(c2)oc(c23)ccc4c3cccc4	AR AF-2	[4]
31	c1cccc2c1ccc(c23)oc(c3)C(=O)N\N=C\c4ccc(cc4)OCCC	AR AF-2	[4]
32	c1ccccc1CCC(=O)C2=C([O-])C(=O)N(Cc3ccccc3)[C@@H]2c4cc(F)ccc4	AR AF-2	[9]
33	c1ccccc1CCC(=O)C2=C([O-])C(=O)N(Cc3ccccc3)[C@H]2c4cc(F)ccc4	AR AF-2	[9]
34	c1cccc(c12)cc([O-])c(c2)C(=O)N\N=C\c3ccccc3	AR AF-2	[10]
35	c1cccc(c12)cc([O-])c(c2)C(=O)N\N=C\c3c(O)ccc(c3)O	AR AF-2	[10]
36	c1cccc(c12)cc([O-])c(c2)C(=O)N\N=C\c3c(O)ccc(c3)OC	AR AF-2	[10]
37	[O-]C(=O)c1cc(c(O)cc1)/C=N/NC(=O)c(c2)c([O-])cc(c23)cccc3	AR AF-2	[10]
38	c1cccc(c12)cc([O-])c(c2)C(=O)N\N=C\c(c([O-])cc3)cc3C(=O)OC	AR AF-2	[10]
39	c1cccc(c12)cc([O-])c(c2)C(=O)N\N=C\c3c([O-])ccc(c3)[N+]([O-])=O	AR AF-2	[10]
40	c1cccc(c12)cc([O-])c(c2)C(=O)N\N=C\c3c(O)c(C)ccc3	AR AF-2	[10]
41	c1cccc(c12)cc([O-])c(c2)C(=O)N\N=C\c3c(O)c(OC)ccc3	AR AF-2	[10]
42	CC(C)(C)OC(=O)NCC(=O)Nc(cc1)c(OCc2ccccc2)cc1C(=O)Nc(cc3)c(cc3C(=O)OCC=C)OCc4ccccc4	AR AF-2	[31]
43	CC(C)COc(c([N+]([O-])=O)cc1)cc1C(=O)Nc(cc2)c(OCC(C)C)cc2C(=O)OC	AR AF-2	[31]
44	CC(=O)c1cc(ccc1)S(=O)(=O)Nc(ccc2)cc2-c3cc[nH]n3	AR AF-2	[32]
45	[O-]C(=O)c1cc(ccc1)CSCc2c(Cl)cc(Cl)cc2	AR BF-3	[6]
46	[O-]C(=O)c1cc(ccc1)CSc2c(Cl)cc(Cl)cc2	AR BF-3	[6]
47	[O-]C(=O)CCSc(n1)n(c(c12)cccc2)CCOc(cc3)ccc3C	AR BF-3	[6]
48	Cc1ccc(cc1)OCCn(c(c23)cccc2)c(n3)SCCOc4ccccc4	AR BF-3	[6]

49	c1cc(O)c(O)cc1C[C@H](C)[C@@H](C)Cc2cc(O)c(O)cc2	AR BF-3	[6]
50	CC(=O)c(cc1)cc2c1N[C@@H]([C@@H]([C@@H]23)CC=C3)c(cc4)ccc4C(=O)OC	AR BF-3	[6]
51	CCOC(=O)c(cc1)cc2c1N[C@@H]([C@H]([C@@H]23)CC=C3)c4ccccc4	AR BF-3	[6]
52	CN(C)S(=O)(=O)c(cc1)cc2c1N[C@@H]([C@H]([C@H]23)CC=C3)c4ccc(Br)cc4	AR BF-3	[6]
53	c1cc(O)c(O)cc1C(=O)CSc([nH]c2=O)nc(c23)cccc3	AR BF-3	[6]
54	[O-]C(=O)/C=C/c1c(C)n(c(C)c1)-c(c2)ccc(c23)OCO3	AR BF-3	[6]
55	CC(C)OC(=O)Cn(c(c12)cccc1)c(n2)SCCOc(cc3C)ccc3	AR BF-3	[11]
56	c1cccc(c12)n(CCOC)c(n2)SCCOc(cc3C)ccc3	AR BF-3	[11]
57	CC(C)OC(=O)Cn(c(c12)cccc1)c(n2)SCCOc(cc3)ccc3C	AR BF-3	[11]
58	CCOC(=O)Cn(c(c12)cccc1)c(n2)SCCOc(c3C)cccc3	AR BF-3	[11]
59	Cc1ccc(cc1)OCCn(c(c23)cccc2)c(n3)SCCOc(cc4)ccc4CC	AR BF-3	[11]
60	c1cccc(c12)n(CCOC)c(n2)SCCOc(cc3)ccc3C	AR BF-3	[11]
61	CC(C)OC(=O)Cn(c(c12)cccc1)c(n2)SCCOc(c3)ccc(C)c3C	AR BF-3	[11]
62	c1cccc(c12)n(CC(=O)OC)c(n2)SCCOc(cc3C)ccc3	AR BF-3	[11]
63	Cc1ccc(cc1)OCCSc(n2)n(CC)c(c23)cccc3	AR BF-3	[11]
64	CCOC(=O)Cn(c(c12)cccc1)c(n2)SCCOc(c3)ccc(C)c3C	AR BF-3	[11]
65	Cc1ccc(cc1)OCCSc(n2)n(C)c(c23)cccc3	AR BF-3	[11]
66	CCOC(=O)Cn(c(c12)cccc1)c(n2)SCCOc(cc3)ccc3CC	AR BF-3	[11]
67	c1cccc(c12)n(CC([O-])=O)c(n2)SCCOc(cc3C)ccc3	AR BF-3	[11]
68	c1ccccc1OCCSc(n2)[nH]c(c23)cccc3	AR BF-3	[11]
69	c1ccccc1OCCSc(n2)n(CC)c(c23)cccc3	AR BF-3	[11]
70	c1ccccc1CCCSc(n2)[nH]c(c23)cccc3	AR BF-3	[11]
71	c1ccccc1CCSc(n2)[nH]c(c23)cccc3	AR BF-3	[11]
72	c1cccc(c1C)OCCSc(n2)[nH]c(c23)cccc3	AR BF-3	[11]
73	Cc1cc(ccc1)OCCSc(n2)[nH]c(c23)cccc3	AR BF-3	[11]
74	Cc1ccc(cc1)OCCSc(n2)[nH]c(c23)cccc3	AR BF-3	[11]
75	c1ccc(C)c(c1C)OCCSc(n2)[nH]c(c23)cccc3	AR BF-3	[11]
76	c1ccc(Cl)cc1OCCSc(n2)[nH]c(c23)cccc3	AR BF-3	[11]
77	c1cc(Cl)ccc1OCCSc(n2)[nH]c(c23)cccc3	AR BF-3	[11]
78	c1cc(S(=O)(=O)N)ccc1OCCSc(n2)[nH]c(c23)cccc3	AR BF-3	[11]
79	c1ccccc1OCCSc(c2)[nH]c(c23)cccc3	AR BF-3	[11]
80	c1ccccc1OCCSc(c2)[nH]c(c23)c(S(=O)(=O)N)ccc3	AR BF-3	[11]
81	c1ccccc1\C=C\c2c[nH]c(c23)cccc3	AR BF-3	[12]
82	c1cccc(c1C(F)(F)F)\N=C\c2c[nH]c(c23)cccc3	AR BF-3	[12]
83	c1cc(Cl)cc(Cl)c1\N=C\c2c[nH]c(c23)cccc3	AR BF-3	[12]
84	Cc1cc(ccc1)\N=C\c2c[nH]c(c23)cccc3	AR BF-3	[12]
85	c1ccc(OC)cc1\N=C\c2c[nH]c(c23)cccc3	AR BF-3	[12]
86	c1cccc(c12)[nH]cc2/C=N/c3cccc(C)c3C	AR BF-3	[12]
87	Clc1cccc(Cl)c1\N=C\c2c[nH]c(c23)cccc3	AR BF-3	[12]
88	c1cccc(OC)c1\N=C\c2c[nH]c(c23)cccc3	AR BF-3	[12]
89	Clc1cccc(c1Cl)\N=C\c2c[nH]c(c23)cccc3	AR BF-3	[12]
90	Brc1cccc(c1Br)\N=C\c2c[nH]c(c23)cccc3	AR BF-3	[12]
91	c1cccc(c1C(F)(F)F)\N=N\c2c[nH]c(c23)cccc3	AR BF-3	[12]
92	c1cccc(c12)[nH]cc2/N=N/c3ccccc3	AR BF-3	[12]
93	c1cccc(c12)[nH]cc2/N=N/c3c(Cl)cccc3	AR BF-3	[12]
94	Cc1c(C)cccc1\N=N\c2c[nH]c(c23)cccc3	AR BF-3	[12]
95	c1cccc(c12)[nH]cc2/N=N/c3cc(Cl)ccc3	AR BF-3	[12]
96	Cc1cc(ccc1)\N=N\c2c[nH]c(c23)cccc3	AR BF-3	[12]
97	c1cccc(c12)[nH]cc2/N=N/c(cccc3)c3-c4ccccc4	AR BF-3	[12]
98	c1cccc(c12)[nH]cc2-c(c3)[nH]c(c34)cccc4	AR BF-3	[12]
99	c1cccc(c12)n(C)c(c2)-c3cn(C)c(c34)cccc4	AR BF-3	[12]

100	c1cccc(c12)[nH]cc2C([C@@H]3N=O)=Nc(c34)cccc4	AR BF-3	[12]
101	c1cccc(c12)[nH]cc2-c(n3)[nH]c(c34)cccc4	AR BF-3	[12]
102	c1cccc(c12)[nH]cc2C(C3)Cc(c34)cccc4	AR BF-3	[12]
103	c1cccc(c12)C[C@H]([C@@H]2C)c3c[nH]c(c34)cccc4	AR BF-3	[12]
104	o1c(Br)ccc1CN(C)C(=O)c(c2)[nH]c(c23)cc(F)cc3	AR BF-3	[7]
105	c1c(F)ccc(c12)[nH]c(c2)C(=O)N(C)Cc3ccc(o3)C	AR BF-3	[7]
106	c1ccccc1CN(CCC#N)C(=O)c(c2)[nH]c(c23)cccc3	AR BF-3	[7]
107	o1cccc1CN(CCOC)C(=O)c(c2)[nH]c(c23)ccc(F)c3	AR BF-3	[7]
108	o1cccc1CN(C(C)C)C(=O)c(c2)[nH]c(c23)ccc(C(F)(F)F)c3	AR BF-3	[7]
109	o1cccc1CN(Cc2ccco2)C(=O)c(c3)[nH]c(c34)cc(F)cc4	AR BF-3	[7]
110	s1c(Br)ccc1CN(C)C(=O)c(c2)[nH]c(c23)cc(F)cc3	AR BF-3	[7]
111	s1cccc1CN(C)C(=O)c(c2)[nH]c(c23)cc(F)cc3	AR BF-3	[7]
112	c1cccc(c12)[nH]c(c2)C(=O)N(C(C)C)Cc3ccc(o3)C	AR BF-3	[7]
113	c1cc(C)cc(c12)[nH]c(c2)C(=O)N(C(C)C)Cc3ccc(o3)C	AR BF-3	[7]
114	Cc(c1)ccc(c12)[nH]c(c2)C(=O)N(C(C)C)Cc3ccc(o3)C	AR BF-3	[7]
115	o1cccc1CN(C(C)C)C(=O)c(c2)[nH]c(c23)ccc(C)c3	AR BF-3	[7]
116	c1c(F)ccc(c12)[nH]c(c2)C(=O)N(C(C)C)Cc3ccc(o3)C	AR BF-3	[7]
117	o1cccc1CN(C(C)C)C(=O)c(c2)[nH]c(c23)cc(C)cc3	AR BF-3	[7]
118	c1ccc(F)c(c12)[nH]c(c2)C(=O)N(C(C)C)Cc3ccc(o3)C	AR BF-3	[7]
119	c1cc(F)cc(c12)[nH]c(c2)C(=O)N(C(C)C)Cc3ccc(o3)C	AR BF-3	[7]
120	o1cccc1CN(C(C)C)C(=O)c(c2)[nH]c(c23)ccc(F)c3	AR BF-3	[7]
121	o1cccc1CN(C(C)C)C(=O)c(c2)[nH]c(c23)cc(F)cc3	AR BF-3	[7]
122	c1ccc(C)c(c12)[nH]cc2-c(cc3)nc(c34)cccc4	AR BF-3	[13]
123	Cc(c1)[nH]c(c12)cccc2	AR BF-3	[3]
124	c1cccc(c1C([O-])=O)Nc(cc2C(F)(F)F)ccc2	AR BF-3	[3]
125	[O-]C(=O)c1c[nH]c(c12)cccc2	AR BF-3	[3]
126	c1cccc(c1C([O-])=O)Nc(c(Cl)c2C)c(Cl)cc2	AR BF-3	[3]
127	[O-]C(=O)[C@@H]([NH3+])Cc(cc1I)cc(I)c1Oc(cc2)cc(I)c2O	AR BF-3	[3]
128	c1cccc(c1C([O-])=O)Nc(c2C)cccc2Cl	AR BF-3	[3]
129	[O-]C(=O)Cc(cc1I)cc(I)c1Oc(cc2)cc(I)c2O	AR BF-3	[3]
130	NC(=O)Cc1cn(CC(C)C)c(c12)ccc(c2)-c3c(OC)cccc3	AR BF-3	[34]
131	s1cccc1C(=O)Nc(n(n2)-c(c3C)cccc3)cc2-c4c(Cl)cccc4	AR BF-3	[34]
132	CC(C)(C)c1cc(ccn1)-c(c2CC(C)C)n(c(=O)c([O-])c2)Cc3ccccc3	ERα AF-2	[14]
133	CC(C)(C)c1cc(ccn1)-c(c2CC(C)C)n(c(=O)c(c2)O)Cc3ccccc3	ERα AF-2	[14]
134	c1cccc(Cl)c1N(C(=O)C)\N=C(\C2=O)Sc(c23)c(C)ccc3	ERα AF-2	[33]
135	C1CCCCC1Cc2c(OCC(=O)OCC)ccc(c2)-c3ccc(cc3)OCC[NH+](C)C	ERα AF-2	[15]
136	CC(C)CNc(cc1CC)nc(n1)NCC(C)C	ERα AF-2	[5]
137	c1ccccc1CNc(n2)nc(NCC(C)C)cc2CCc3ccccc3	ERα AF-2	[5]
138	CC(C)CCc1cc(NCC(C)C)nc(n1)NCC(C)C	ERα AF-2	[5]
139	CC(C)CNC(=N1)NC(CCC(C)C)C=C1NCc2ccccc2	ERα AF-2	[5]
140	c1ccccc1CCC(NC(=N2)NCC(C)C)C=C2NCc3ccccc3	ERα AF-2	[5]
141	CC(C)CNc(n1)nc(NCC(C)C)cc1CCc2cccc(c23)cccc3	ERα AF-2	[5]
142	c1ccccc1CCc2cc(NCC(C)C)nc(n2)NCc3cccc(c34)cccc4	ERα AF-2	[5]
143	c1ccccc1CNC2=CC(NC(=N2)NCC(C)C)CCc3cccc(c34)cccc4	ERα AF-2	[5]
144	COC(=O)C(C(=O)OC)=C[C@](C)([C@@H]12)C[C@H](C=C1)[C@H](C2)C(C)(C)CCNC(=O)CCC(C)C	ERα AF-2	[30]
145	COC(=O)C(C(=O)OC)C[C@](C)([C@H]12)C[C@H](CC2)[C@H](C1)C(C)(C)CCNC(=O)CCC(C)C	ERα AF-2	[30]
146	COC(=O)[C@@H](C([O-])=O)C[C@](C)([C@H]12)C[C@H](CC2)[C@H](C1)C(C)(C)CCNC(=O)CCC(C)C	ERα AF-2	[30]
147	c1ccccc1COC(=O)N[C@H](C(=O)OC)C[C@](C)([C@H]23)C[C@H](CC3)[C@H](C2)C(C)(C)CCNC(=O)CCC(C)C	ERα AF-2	[30]
148	COC(=O)[C@@H](N)C[C@](C)([C@H]12)C[C@H](CC2)[C@H](C1)C(C)(C)CCNC(=O)CCC(C)C	ERα AF-2	[30]
149	CCCCc1c(CC[NH3+])c(CCCC)c(CC[NH3+])c(CCCC)c1CC[NH3+]	ERα AF-2	[8]
150	CCCCCc1c(CC[NH3+])c(CCCCC)c(CC[NH3+])c(c1CC[NH3+])CCCCC	ERα AF-2	[8]

151	CCCCCCc1c(CC[NH3+])c(CCCCCC)c(CC[NH3+])c(c1CC[NH3+])CCCCCC	ERα AF-2	[8]
152	CC(C)(C)CCc1c(CC[NH3+])c(CCC(C)(C)C)c(CC[NH3+])c(CCC(C)(C)C)c1CC[NH3+]	ERα AF-2	[8]
153	c1ccccc1/C=C/c2cc(NCC(C)C)nc(n2)NCC(C)C	ERα AF-2	[5]
154	CC(C)CNc(n1)nc(NCC(C)C)cc1CCc2ccccc2	ERα AF-2	[5]
155	c1ccccc1/C=C/C(NC(=N2)NCC(C)C)C=C2NCc3ccccc3	ERα AF-2	[5]
156	c1ccccc1CNc(n2)nc(NCC(C)C)cc2CCc3ccccc3	ERα AF-2	[5]
157	c1ccccc1CCC(NC(=N2)NCC(C)C)C=C2NCc3ccccc3	ERα AF-2	[5]
158	CCCCc1cc(NCCC)nc(n1)NCCC	ERα AF-2	[5]
159	CC(C)CCc1cc(NCC(C)C)nc(n1)NCC(C)C	ERα AF-2	[5]
160	c1ccccc1CNC(=N2)NC(CCC(C)C)C=C2NCc3ccccc3	ERα AF-2	[5]
161	CC(C)CNc(n1)nc(NCC(C)C)cc1CCc2cccc(c23)cccc3	ERα AF-2	[5]
162	CC(C)CNc(cc1CC)nc(n1)NCC(C)C	ERα AF-2	[5]
163	CC(C)CCc1cc(NCC(C)C)nc(n1)NCc2ccccc2	ERα AF-2	[5]
164	CC(C)CCc1cc(NCC(C)C)nc(n1)NCc2cccc(c23)cccc3	ERα AF-2	[5]
165	CC(C)CNC(=N1)NC(CCC(C)C)C=C1NCc2ccccc2	ERα AF-2	[5]
166	CC(C)CNC(=N1)NC(CCC(C)C)C=C1NCc2cccc(c23)cccc3	ERα AF-2	[5]
167	CC(C)CNc1cc(NCC(C)C)nc(n1)NCC(C)C	ERα AF-2	[5]
168	CC(C)CNc1cc(nc(n1)NCC(C)C)NCc2ccccc2	ERα AF-2	[5]
169	CC(C)CCc1cc(NCC(C)C)nc(n1)N(C)CC(C)C	ERα AF-2	[5]
170	CC(C)CNc(n1)nc(N(C)CC(C)C)cc1CCc2cccc(c23)cccc3	ERα AF-2	[5]
171	CC(C)CNc(n1)nc(N(C)CC(C)C)cc1CCc2ccccc2	ERα AF-2	[5]
172	CC(C)CCc1cc(NCC(C)C)nc(n1)NCC(C)C	ERα AF-2	[16]
173	CCCCc1cc(NCCC)nc(n1)NCCC	ERα AF-2	[16]
174	c1ccccc1CCC2=CC(NCc3ccccc3)=NC(N2)NCc4ccccc4	ERα AF-2	[16]
175	Clc1c(Cl)ccc(c1)N(CC2)CCN2C(=O)CCCn(c3=O)c(=S)[nH]c(c34)cccc4	ERα AF-2	[17]
176	c1cccc(O)c1N(CC2)CCN2C(=O)CCCn(c3=O)c(=S)[nH]c(c34)cccc4	ERα AF-2	[17]
177	CC(C)NC(=O)CSc(n1)sc(c12)cc(cc2)NC(=O)CSc3cc(Cl)ccc3	ERα AF-2	[17]
178	NC(N)=[NH+]\N=C\C(=C1Cl)CCc(c12)cccc2	ERα AF-2	[18]
179	NC(N)=[NH+]\N=C\C(=C1Cl)CCc(c12)ccc(c2)OC	ERα AF-2	[18]
180	NC(N)=[NH+]\N=C\C(=C1Cl)CCc(c12)cc(cc2)OC	ERα AF-2	[18]
181	NC(N)=[NH+]\N=C\C(=C1Cl)CCc(c12)c(C)cc(C)c2	ERα AF-2	[18]
182	NC(N)=[NH+]\N=C\C(=C1Cl)C[C@@H](C)c(c12)cccc2	ERα AF-2	[18]
183	NC(N)=[NH+]\N=C\C(=C1Cl)Cc(c12)cccc2	ERα AF-2	[18]
184	NC(N)=[NH+]\N=C\C(=C1Cl)CCCc(c12)cccc2	ERα AF-2	[18]
185	NC(N)=[NH+]\N=C\C(=C1Br)CCc(c12)cccc2	ERα AF-2	[18]
186	c1cccc(c12)CCC(/C=N/[NH+]=C(N)N)=C2Oc3ccccc3	ERα AF-2	[18]
187	NC(N)=[NH+]\N=C\C=C(Cl)\c1ccccc1	ERα AF-2	[18]
188	NC(N)=[NH+]/N=C/C(C)=C(Cl)/c1ccccc1	ERα AF-2	[18]
189	NC(N)=[NH+]\N=C\c1cccc(c12)cccc2	ERα AF-2	[18]
190	NC(N)=[NH+]\N=C\c(c1)ccc(c12)cccc2	ERα AF-2	[18]
191	CC[C@@H](C)c1c(OCC([O-])=O)ccc(c1)-c(c2)ccc(c2[C@@H](C)CC)OCC[NH+](C)C	ERα AF-2	[19]
192	CC[C@@H](C)c1c(OCC([O-])=O)ccc(c1)-c(c2)ccc(c2[C@H](C)CC)OCC[NH+](C)C	ERα AF-2	[19]
193	CC[C@H](C)c1c(OCC([O-])=O)ccc(c1)-c(c2)ccc(c2[C@@H](C)CC)OCC[NH+](C)C	ERα AF-2	[19]
194	CC[C@H](C)c1c(OCC([O-])=O)ccc(c1)-c(c2)ccc(c2[C@H](C)CC)OCC[NH+](C)C	ERα AF-2	[19]
195	NC(N)=[NH+]\N=C\C(=C1Cl)CCc(c12)cccc2	ERα AF-2	[20]
196	C[NH+](C)CCOC(=O)Nc(cc1)ccc1Cc2ccc(cc2)NC(=O)OCC[NH+](C)C	ERα AF-2	[20]
197	c1ccccc1C(\CC)=C(c2ccc(O)cc2)/c3ccc(cc3)OCC[NH+](C)C	ERβ AF-2	[21]
198	c1ccccc1/C=C/c2cc(NCC(C)C)nc(n2)NCC(C)C	ERβ AF-2	[5]
199	c1ccccc1CN(C(=O)OC(C)(C)C)c(nc(n2)NCC(C)C)cc2\C=C\c3ccccc3	ERβ AF-2	[5]
200	C=CC(=O)c1ccc(cc1)CCCCCC	TRα AF-2	[22]
201	CCCCCCc(cc1)ccc1C(=O)CC[N@H+](C)CCc2ccccc2	TRα AF-2	[23]

202	CCCCCCc(cc1)ccc1C(=O)CC[N@H+](C)CCc2ccccc2	TRα AF-2	[23]
203	CC(C)[NH+](C(C)C)CCC(=O)c1ccc(cc1)CCCCCC	TRα AF-2	[23]
204	C1CC=CN1CCC(=O)c2ccc(cc2)CCCCCC	TRα AF-2	[23]
205	C1COCCN1CCC(=O)c2ccc(cc2)CCCCCC	TRα AF-2	[23]
206	CCCC[NH+](CCCC)CCC(=O)c1ccc(cc1)CCCCCC	TRα AF-2	[23]
207	C[NH+](C)CCC(=O)c1ccc(cc1)CCCCCC	TRα AF-2	[23]
208	C1CCCCC1[NH+](C2CCCCC2)CCC(=O)c3ccc(cc3)CCCCCC	TRα AF-2	[23]
209	CCC[NH2+]CCC(=O)c1ccc(cc1)CCCCCC	TRα AF-2	[23]
210	[O-]C(=O)/C=C\C(=O)c1ccc(cc1)CCCCCC	TRα AF-2	[23]
211	C\C=C\C(=O)c1ccc(cc1)CCCCCCC	TRα AF-2	[23]
212	C=C(C)C(=O)c1ccc(cc1)CCCCCCC	TRα AF-2	[23]
213	C=CC(=O)c1ccc(cc1)CCCC	TRα AF-2	[23]
214	C=CC(=O)c1ccc(cc1)CCCCC	TRα AF-2	[23]
215	C=CC(=O)c1ccc(cc1)CC(C)(C)C	TRα AF-2	[23]
216	C=CC(=O)c1ccc(cc1)CCCCCCC	TRα AF-2	[23]
217	C=CC(=O)c1ccc(cc1)CCCCCCCC	TRα AF-2	[23]
218	C=CC(=O)[C@H]1CC[C@@H](CC1)CC(C)(C)C	TRα AF-2	[23]
219	C=CC(=O)OC(=O)c1c(cccc1)CCCCCC	TRα AF-2	[23]
220	C=CC(=O)Nc(cc1)ccc1CCCCCC	TRα AF-2	[23]
221	[O-]C(=O)/C=C\C(=O)Nc(cc1)ccc1CCCCCC	TRα AF-2	[23]
222	C\C=C\C(=O)Nc(cc1)ccc1CCCCCC	TRα AF-2	[23]
223	C#CC(=O)Nc(cc1)ccc1CCCCCC	TRα AF-2	[23]
224	C#CC(=O)Oc(cc1)ccc1CCCCCC	TRα AF-2	[23]
225	ClCC(=O)c1ccc(cc1)CCCCCC	TRα AF-2	[23]
226	BrCCC(=O)c1ccc(cc1)CCCCCC	TRα AF-2	[23]
227	O1C[C@@H]1C(=O)c2ccc(cc2)CCCCCC	TRα AF-2	[23]
228	C=CC(=O)c1ccc(cc1)CCCCCC	TRβ AF-2	[22]
229	CCCCCCc(cc1)ccc1C(=O)CC[N@H+](C)CCc2ccccc2	TRβ AF-2	[23]
230	CCCCCCc1ccc(C(=O)CCN(C)CCC)cc1	TRβ AF-2	[23]
231	CC(C)[NH+](C(C)C)CCC(=O)c1ccc(cc1)CCCCCC	TRβ AF-2	[23]
232	C1CC=CN1CCC(=O)c2ccc(cc2)CCCCCC	TRβ AF-2	[23]
233	C1COCCN1CCC(=O)c2ccc(cc2)CCCCCC	TRβ AF-2	[23]
234	CCCC[NH+](CCCC)CCC(=O)c1ccc(cc1)CCCCCC	TRβ AF-2	[23]
235	C[NH+](C)CCC(=O)c1ccc(cc1)CCCCCC	TRβ AF-2	[23]
236	C1CCCCC1[NH+](C2CCCCC2)CCC(=O)c3ccc(cc3)CCCCCC	TRβ AF-2	[23]
237	CCC[NH2+]CCC(=O)c1ccc(cc1)CCCCCC	TRβ AF-2	[23]
238	[O-]C(=O)/C=C\C(=O)c1ccc(cc1)CCCCCC	TRβ AF-2	[23]
239	C\C=C\C(=O)c1ccc(cc1)CCCCCCC	TRβ AF-2	[23]
240	C=C(C)C(=O)c1ccc(cc1)CCCCCCC	TRβ AF-2	[23]
241	C=CC(=O)c1ccc(cc1)CCC	TRβ AF-2	[23]
242	C=CC(=O)c1ccc(cc1)CCCC	TRβ AF-2	[23]
243	C=CC(=O)c1ccc(cc1)CCCCC	TRβ AF-2	[23]
244	C=CC(=O)c1ccc(cc1)CC(C)(C)C	TRβ AF-2	[23]
245	C=CC(=O)c1ccc(cc1)CCCCCCC	TRβ AF-2	[23]
246	C=CC(=O)c1ccc(cc1)CCCCCCCC	TRβ AF-2	[23]
247	C=CC(=O)[C@H]1CC[C@@H](CC1)CC(C)(C)C	TRβ AF-2	[23]
248	C=CC(=O)OC(=O)c1ccc(cc1)CCC	TRβ AF-2	[23]
249	C=CC(=O)OC(=O)c1ccc(cc1)CCCCC	TRβ AF-2	[23]
250	C=CC(=O)OC(=O)c1c(cccc1)CCCCCC	TRβ AF-2	[23]
251	C=CC(=O)c1ccc(cc1)NC(=O)CCC	TRβ AF-2	[23]
252	C=CC(=O)c(c1)ccc(c12)CCCC2	TRβ AF-2	[23]

253	C=CC(=O)Nc(cc1)ccc1CCCCCC	TRβ AF-2	[23]
254	[O-]C(=O)/C=C\C(=O)Nc(cc1)ccc1CCCCCC	TRβ AF-2	[23]
255	C\C=C\C(=O)Nc(cc1)ccc1CCCCCC	TRβ AF-2	[23]
256	C#CC(=O)Nc(cc1)ccc1CCCCCC	TRβ AF-2	[23]
257	C#CC(=O)Oc(cc1)ccc1CCCCCC	TRβ AF-2	[23]
258	ClCC(=O)c1ccc(cc1)CCCCCC	TRβ AF-2	[23]
259	BrCCC(=O)c1ccc(cc1)CCCCCC	TRβ AF-2	[23]
260	O1C[C@@H]1C(=O)c2ccc(cc2)CCCCCC	TRβ AF-2	[23]
261	C[NH+](C)CCC(=O)c1ccc(cc1)OCCCCCC	TRβ AF-2	[24]
262	C[NH+](C)CCC(=O)c1cc(ccc1)OCCCCCC	TRβ AF-2	[24]
263	C[NH+](C)CCC(=O)c1ccc(c(c12)cccc2)OCCCCCC	TRβ AF-2	[24]
264	CCCCCCOc(cc1)cc(c12)C[C@H](C2=O)C[NH+](C)C	TRβ AF-2	[24]
265	CCCCCCOc(cc1)cc(c12)C[C@@H](C2=O)C[NH+](C)C	TRβ AF-2	[24]
266	CCCCCCOc(cc1)cc(c12)CC[C@@H](C2=O)C[NH+](C)C	TRβ AF-2	[24]
267	CCCCCCOc(cc1)cc(c12)CC[C@H](C2=O)C[NH+](C)C	TRβ AF-2	[24]
268	CCCCCCOc(cc1)cc(c12)OC[C@@H](C2=O)C[NH+](C)C	TRβ AF-2	[24]
269	CCCCCCOc(cc1)cc(c12)OC[C@@H](C2=O)CN(C)C	TRβ AF-2	[24]
270	CCCC[NH+](CCCC)CCC(=O)c1ccc(cc1)OCCCCCC	TRβ AF-2	[24]
271	C1CCCC[NH+]1CCC(=O)c2ccc(cc2)OCCCCCC	TRβ AF-2	[24]
272	C1CCC[NH+]1CCC(=O)c2ccc(cc2)OCCCCCC	TRβ AF-2	[24]
273	C[C@@H]1C[N@H+]1CCC(=O)c2ccc(cc2)OCCCCCC	TRβ AF-2	[24]
274	C1CN(C)CCN1CCC(=O)c2ccc(cc2)OCCCCCC	TRβ AF-2	[24]
275	C1CN(C)CCN1CCC(=O)c2ccc(cc2)OCCCCCC	TRβ AF-2	[24]
276	CCCCCCOc(cc1)ccc1C(=O)CCN2CCN(CC2)c3ccccc3	TRβ AF-2	[24]
277	CCCCCCOc(cc1)ccc1C(=O)CC[NH+](CC2)CCN2c3ccccc3	TRβ AF-2	[24]
278	C1COCCN1CCC(=O)c2ccc(cc2)OCCCCCC	TRβ AF-2	[24]
279	C[NH+](C)CCC(=O)c1ccc(cc1)SCCCCCC	TRβ AF-2	[24]
280	C[NH+](C)CCC(=O)c1ccc(cc1)S(=O)(=O)CCCCCC	TRβ AF-2	[24]
281	C[NH+](C)CCC(=O)c1ccc(cc1)C(=O)NCCCCCC	TRβ AF-2	[24]
282	C[NH+](C)CCC(=O)c1ccc(cc1)NC(=O)CCCCC	TRβ AF-2	[24]
283	CCCCCCS(=O)(=O)c2ccc(C(=O)CCN1CCNC(=O)C1)c(Cl)c2Cl	TRβ AF-2	[24]
284	CCCCCCS(=O)(=O)c2ccc(C(=O)CCN1CCN(C(C)=O)C(=O)C1)c(Cl)c2Cl	TRβ AF-2	[24]
285	CCCCCCS(=O)(=O)c2cc(Cl)c(C(=O)CCN1CCNC(=O)C1)cc2Cl	TRβ AF-2	[24]
286	CCCCCCS(=O)(=O)c2cc(Cl)c(C(=O)CCN1CCN(C(C)=O)C(=O)C1)cc2Cl	TRβ AF-2	[24]
287	C1[C@H](C2)C[C@@H](C3)C[C@H]2CC13NC(=O)COC(=O)c4cc([N+]([O-])=O)c(cc4)S(=O)(=O)C	TRβ AF-2	[25]
288	C1CCCCC1C(=O)NC(=O)COC(=O)c2cc([N+]([O-])=O)c(cc2)S(=O)(=O)C	TRβ AF-2	[25]
289	CN3CC[C@]2(C)c1ccccc1[N@](C)C2O3	TRβ AF-2	[25]
290	C1CCCCC1NC(=O)NC(=O)COC(=O)c2cc([N+]([O-])=O)c(cc2)S(=O)(=O)C	TRβ AF-2	[26]
291	Cn1cccc1C(=O)NC(=O)COC(=O)c2cc([N+]([O-])=O)c(cc2)S(=O)(=O)C	TRβ AF-2	[26]
292	C[C@@]12C(C)(C)[C@H](CC2)[C@@H](C1)NC(=O)COC(=O)c3cc([N+]([O-])=O)c(cc3)S(=O)(=O)C	TRβ AF-2	[26]
293	CC1CCN(CC1)C(=O)[C@H](C)OC(=O)c2cc([N+]([O-])=O)c(cc2)S(=O)(=O)C	TRβ AF-2	[26]
294	CC1CCN(CC1)C(=O)[C@@H](C)OC(=O)c2cc([N+]([O-])=O)c(cc2)S(=O)(=O)C	TRβ AF-2	[26]
295	C[C@@H]1CCCCN1C(=O)COC(=O)c2cc([N+]([O-])=O)c(cc2)S(=O)(=O)C	TRβ AF-2	[26]
296	C[C@H]1CCCCN1C(=O)COC(=O)c2cc([N+]([O-])=O)c(cc2)S(=O)(=O)C	TRβ AF-2	[26]
297	C1CCCN1C(=O)COC(=O)c2cc([N+]([O-])=O)c(cc2)S(=O)(=O)C	TRβ AF-2	[26]
298	c1cccc(c12)NC(=O)CN2C(=O)COC(=O)c3cc([N+]([O-])=O)c(cc3)S(=O)(=O)C	TRβ AF-2	[26]
299	c1cccc(c12)N(CC2)C(=O)COC(=O)c3cc([N+]([O-])=O)c(cc3)S(=O)(=O)C	TRβ AF-2	[26]
300	CC(=O)c1cc(ccc1)NC(=O)[C@H](C)OC(=O)c2cc([N+]([O-])=O)c(cc2)S(=O)(=O)C	TRβ AF-2	[26]
301	CC(=O)c1cc(ccc1)NC(=O)[C@@H](C)OC(=O)c2cc([N+]([O-])=O)c(cc2)S(=O)(=O)C	TRβ AF-2	[26]
302	C1[C@@H](C2)C[C@H](C3)C[C@@H]2[C@H]([C@@H]13)NC(=O)COC(=O)c4cc([N+]([O-])=O)c(cc4)S(=O)(=O)C	TRβ AF-2	[26]
303	C1[C@@H](C2)C[C@H](C3)C[C@@H]2[C@H]([C@@H]13)NC(=O)COC(=O)c4cc([N+]([O-])=O)c(F)cc4	TRβ AF-2	[26]

304	C1[C@H](C2)C[C@@H](C3)C[C@H]2CC13NC(=O)c4cnc(s4)-c5cc([N+]([O-])=O)c(cc5)S(=O)(=O)C	TRβ AF-2	[27]
305	C1CCCCN1C(=O)c2cnc(s2)-c3cc([N+]([O-])=O)c(cc3)S(=O)(=O)C	TRβ AF-2	[27]
306	C[C@@]12C[C@H](CC(C)(C)C2)N(C1)C(=O)c3c(C)nc(s3)-c4cc([N+]([O-])=O)c(cc4)S(=O)(=O)C	TRβ AF-2	[27]
307	C1CCCCN1C(=O)c2c(C)nc(s2)-c3cc([N+]([O-])=O)c(cc3)S(=O)(=O)C	TRβ AF-2	[27]
308	CC1CCN(CC1)C(=O)c2c(C)nc(s2)-c3cc([N+]([O-])=O)c(cc3)S(=O)(=O)C	TRβ AF-2	[27]
309	C[C@]12C[C@@H](CC(C)(C)C2)N(C1)C(=O)c3c(CC)nc(s3)-c4cc([N+]([O-])=O)c(cc4)S(=O)(=O)C	TRβ AF-2	[27]
310	C[C@@]12C[C@H](CC(C)(C)C2)N(C1)C(=O)c3c(CC)nc(s3)-c4cc([N+]([O-])=O)c(cc4)S(=O)(=O)C	TRβ AF-2	[27]
311	C1CCCCN1C(=O)c2c(CC)nc(s2)-c3cc([N+]([O-])=O)c(cc3)S(=O)(=O)C	TRβ AF-2	[27]
312	C[C@]12C[C@@H](CC(C)(C)C2)N(C1)C(=O)c(c3C(F)(F)F)sc(n3)-c4cc([N+]([O-])=O)c(cc4)S(=O)(=O)C	TRβ AF-2	[27]
313	C[C@@]12C[C@H](CC(C)(C)C2)N(C1)C(=O)c(c3C(F)(F)F)sc(n3)-c4cc([N+]([O-])=O)c(cc4)S(=O)(=O)C	TRβ AF-2	[27]
314	C1CCCCN1C(=O)c(c2C(F)(F)F)sc(n2)-c3cc([N+]([O-])=O)c(cc3)S(=O)(=O)C	TRβ AF-2	[27]
315	CC1CCN(CC1)C(=O)c(c2C(F)(F)F)sc(n2)-c3cc([N+]([O-])=O)c(cc3)S(=O)(=O)C	TRβ AF-2	[27]
316	c1cccc(c12)N([C@H](C)C2)C(=O)c(c3C(F)(F)F)sc(n3)-c4cc([N+]([O-])=O)c(cc4)S(=O)(=O)C	TRβ AF-2	[27]
317	c1cccc(c12)N([C@@H](C)C2)C(=O)c(c3C(F)(F)F)sc(n3)-c4cc([N+]([O-])=O)c(cc4)S(=O)(=O)C	TRβ AF-2	[27]
318	C1CCCCN1C(=O)c2c(-c3ccccc3)nc(s2)-c4cc([N+]([O-])=O)c(cc4)S(=O)(=O)C	TRβ AF-2	[27]
319	c1ccc(F)cc1CN(C)C(=O)c2cnc(s2)-c3cc([N+]([O-])=O)c(cc3)S(=O)(=O)C	TRβ AF-2	[27]
320	c1cccc(c12)CN(CC2)C(=O)c(c3C(F)(F)F)sc(n3)-c4cc([N+]([O-])=O)c(cc4)S(=O)(=O)C	TRβ AF-2	[27]
321	c1ccccc1CN(C)C(=O)c(c2C(F)(F)F)sc(n2)-c3cc([N+]([O-])=O)c(cc3)S(=O)(=O)C	TRβ AF-2	[27]
322	c1cccc(F)c1CN(C)C(=O)c(c2C(F)(F)F)sc(n2)-c3cc([N+]([O-])=O)c(cc3)S(=O)(=O)C	TRβ AF-2	[27]
323	c1ccc(F)cc1CN(C)C(=O)c(c2C(F)(F)F)sc(n2)-c3cc([N+]([O-])=O)c(cc3)S(=O)(=O)C	TRβ AF-2	[27]
324	c1ccccc1N(CCC)C(=O)c2csc(n2)-c3cc([N+]([O-])=O)c(cc3)S(=O)(=O)C	TRβ AF-2	[27]
325	C=CC(=O)c1ccc(cc1)CCCCCC	TRβ AF-2	[28]
326	CCC(=O)c1ccc(cc1)CCCCCC	TRβ AF-2	[28]
327	[NH3+]Cc1ccc(cc1)-c(n2)cn3c2sc(c34)cccc4	GR AF_2	[29]
328	[NH3+]Cc1ccc(cc1)-c(n2)cn3c2sc(c34)CCCC4	GR AF_2	[29]
329	OCc1ccc(cc1)-c(n2)cn3c2sc(c34)cccc4	GR AF_2	[29]
330	Cc1ccc(cc1)-c(n2)cn3c2sc(c34)cccc4	GR AF_2	[29]
331	N#Cc1ccc(cc1)-c(c2)nc(n3CC)n2c(c34)cccc4	GR AF_2	[29]
332	c1cccc(c12)sc3n2cc(n3)-c4ccc(N)cc4	GR AF_2	[29]
333	c1cccc(c12)sc3n2cc(n3)-c4ccc(Br)cc4	GR AF_2	[29]
334	N#Cc1ccc(cc1)-c(n2)cn3c2sc(c34)CCCC4	GR AF_2	[29]

[a]SMILES code exported from Maestro.
[b]Reference order in supporting information.

Protein Preparation

Table S21. Protein structures used in this study.

Receptor	Crystal structures MD	Crystal structures Docking	Template structures	UniProt entry
AR	3L3X	2PIT	n/a	P10275
ERα	5WGD	3UUD	1X7R	P03372
ERβ	4J24	2J7Y	3OLS	Q92731
GR	5NFP	3K22	n/a	P04150
MR	2AA2	2AA2	2A3I	P08235
PR	1A28	1A28	n/a	P06401
TRα	4LNW	4LNW	n/a	P10827
TRβ	1XZX	3GWS	2J4A	P10828

[a]Structures that were used to complete missing loops. For several receptors, no additional structures were used.

MD Simulations

Table S22. Conventional Desmond relaxation protocol.

Desmond stage	Procedure
1	Task (reading files, initializing parameters)
2	Simulate, Brownian Dynamics, NVT, T = 10 K, small time steps, and restraints on solute heavy atoms, 100 ps
3	Simulate, NVT, T = 10 K, small time steps, and restraints on solute heavy atoms, 12 ps
4	Simulate, NPT, T = 10 K, and restraints on solute heavy atoms, 12 ps
5	Solvate pocket
6	Simulate, NPT and restraints on solute heavy atoms, 12 ps
7	Simulate, NPT and no restraints, 24 ps

The information about this default relaxation protocol was adapted from our previous work[1].

Table S23. Mixed Solvent MD Desmond relaxation protocol.

Desmond stage	Procedure
1	Brownian Dynamics, NVT, T = 10 K, 1 fs timestep, and restraints on all solute atoms, 24 ps
2	Brownian Dynamics, NVT, T = 10 K, 1 fs timestep, and restraints on solute heavy atoms, 24 ps
3	NVT, T = 10 K, 1 fs timestep, restraints on solute heavy atoms, 12 ps
4	NPT, T = 10 K, 2 fs timestep, restraints on solute heavy atoms, 12 ps
5	NPT, T= 300 K, 2 fs timestep, restraints on solute heavy atoms, 24 ps
6	NPT, T = 300 K, 2 fs timestep, 15 ps

The information about this default relaxation protocol was retrieved from the program documentation.

Crystal Structure Analysis

Table S24. Number input structures and minimal amount for cluster to be considered as conserved.

Receptor	Number of input structures	Minimal occupancy
AR	67	7
ERα	229	23
ERβ	32	3
GR	20	2
MR	27	3
PR	16	2
TRα	8	1
TRβ	9	1

References

1. Fischer, A. & Smieško, M. Spontaneous Ligand Access Events to Membrane-Bound Cytochrome P450 2D6 Sampled at Atomic Resolution. *Sci. Rep.* **9**, 16411 (2019).
2. Munuganti, R. S. N. *et al.* Targeting the binding function 3 (BF3) site of the androgen receptor through virtual screening. 2. Development of 2-((2-phenoxyethyl) thio)-1H-benzimidazole derivatives. *J. Med. Chem.* **56**, 1136–1148 (2013).
3. Axerio-Cilies, P. *et al.* Inhibitors of androgen receptor activation function-2 (AF2) site identified through virtual screening. *J. Med. Chem.* **54**, 6197–6205 (2011).
4. Estebanez-Perpina, E. *et al.* A surface on the androgen receptor that allosterically regulates coactivator binding. *Proc. Natl. Acad. Sci.* **104**, 16074–16079 (2007).
5. Caboni, L. *et al.* 'True' antiandrogens-selective non-ligand-binding pocket disruptors of androgen receptor-coactivator interactions: Novel tools for prostate cancer. *J. Med. Chem.* **55**, 1635–1644 (2012).
6. Gunther, J. R., Parent, A. A. & Katzenellenbogen, J. A. Alternative inhibition of androgen receptor signaling: Peptidomimetic

pyrimidines as direct androgen receptor/coactivator disruptors. *ACS Chem. Biol.* **4**, 435–440 (2009).
7. Lack, N. A. *et al.* Targeting the binding function 3 (BF3) site of the human androgen receptor through virtual screening. *J. Med. Chem.* **54**, 8563–8573 (2011).
8. Ban, F. *et al.* Discovery of 1 H-indole-2-carboxamides as novel inhibitors of the androgen receptor binding function 3 (BF3). *J. Med. Chem.* **57**, 6867–6872 (2014).
9. Parent, A. A., Gunther, J. R. & Katzenellenbogen, J. A. Blocking estrogen signaling after the hormone: pyrimidine-core inhibitors of estrogen receptor-coactivator binding. *J. Med. Chem.* **51**, 6512–6530 (2008).
10. Caboni, L. *et al.* Structure-activity relationships in non-ligand binding pocket (non-LBP) diarylhydrazide antiandrogens. *J. Chem. Inf. Model.* **53**, 2116–2130 (2013).
11. Caboni, L. *et al.* Molecular topology applied to the discovery of 1-benzyl-2-(3-fluorophenyl)-4-hydroxy-3-(3-phenylpropanoyl)-2 h -pyrrole-5-one as a non-ligand-binding-pocket antiandrogen. *J. Chem. Inf. Model.* **54**, 2953–2966 (2014).
12. Munuganti, R. S. N. *et al.* Targeting the binding function 3 (BF3) site of the androgen receptor through virtual screening. 2. Development of 2-((2-phenoxyethyl) thio)-1H-benzimidazole derivatives. *J. Med. Chem.* **56**, 1136–1148 (2013).
13. Munuganti, R. S. N. *et al.* Identification of a Potent Antiandrogen that Targets the BF3 Site of the Androgen Receptor and Inhibits Enzalutamide-Resistant Prostate Cancer. *Chem. Biol.* **21**, 1476–1485 (2014).
14. Lallous, N. *et al.* Targeting Binding Function-3 of the Androgen Receptor Blocks Its Co-Chaperone Interactions, Nuclear Translocation, and Activation. *Mol. Cancer Ther.* **15**, 2936–2945 (2016).
15. Becerril, J. & Hamilton, A. D. Helix mimetics as inhibitors of the interaction of the estrogen receptor with coactivator peptides. *Angew. Chemie - Int. Ed.* **46**, 4471–4473 (2007).
16. Weiser, P. T., Chang, C.-Y., McDonnell, D. P. & Hanson, R. N. 4,4'-Unsymmetrically substituted 3,3'-biphenyl alpha helical proteomimetics as potential coactivator binding inhibitors. *Bioorg. Med. Chem.* **22**, 917–926 (2014).
17. Rodriguez, A. L., Tamrazi, A., Collins, M. L. & Katzenellenbogen, J. A. Design, Synthesis, and in Vitro Biological Evaluation of Small Molecule Inhibitors of Estrogen Receptor α Coactivator Binding. *J. Med. Chem.* **47**, 600–611 (2004).
18. Sun, A. *et al.* Discovering small-molecule estrogen receptor α/coactivator binding inhibitors: high-throughput screening, ligand development, and models for enhanced potency. *ChemMedChem* **6**, 654–666 (2011).
19. LaFrate, A. L., Gunther, J. R., Carlson, K. E. & Katzenellenbogen, J. A. Synthesis and biological evaluation of guanylhydrazone coactivator binding inhibitors for the estrogen receptor. *Bioorganic Med. Chem.* **16**, 10075–10084 (2008).
20. Williams, A. B., Weiser, P. T., Hanson, R. N., Günther, J. R. & Katzenellenbogen, J. A. Synthesis of biphenyl proteomimetics as estrogen receptor-a coactivator binding inhibitors. *Org. Lett.* **11**, 5370–5373 (2009).
21. Shao, D. *et al.* Identification of novel estrogen receptor α antagonists. *J. Steroid Biochem. Mol. Biol.* **88**, 351–360 (2004).
22. Wang, Y. *et al.* A second binding site for hydroxytamoxifen within the coactivator-binding groove of estrogen receptor β. *Proc. Natl. Acad. Sci. U. S. A.* **103**, 9908–9911 (2006).
23. Arnold, L. A. *et al.* Discovery of small molecule inhibitors of the interaction of the thyroid hormone receptor with transcriptional coregulators. *J. Biol. Chem.* **280**, 43048–43055 (2005).
24. Arnold, L. A., Kosinski, A., Estébanez-Perpiñá, E., Fletterick, R. J. & Guy, R. K. Inhibitors of the interaction of a thyroid hormone receptor and coactivators: Preliminary structure-activity relationships. *J. Med. Chem.* **50**, 5269–5280 (2007).
25. Jong, Y. H. *et al.* Improvement of pharmacological properties of irreversible thyroid receptor coactivator binding inhibitors. *J. Med. Chem.* **52**, 3892–3901 (2009).
26. Johnson, R. L. *et al.* A quantitative high-throughput screen identifies novel inhibitors of the interaction of thyroid receptor β with a peptide of steroid receptor coactivator 2. *J. Biomol. Screen.* **16**, 618–627 (2011).
27. Hwang, J. Y. *et al.* Methylsulfonylnitrobenzoates, a new class of irreversible inhibitors of the interaction of the thyroid hormone receptor and its obligate coactivators that functionally antagonizes thyroid hormone. *J. Biol. Chem.* **286**, 11895–11908 (2011).
28. Hwang, J. Y. *et al.* Synthesis and evaluation of sulfonylnitrophenylthiazoles (SNPTs) as thyroid hormone receptor-coactivator interaction inhibitors. *J. Med. Chem.* **55**, 2301–2310 (2012).
29. Estébanez-Perpiñá, E. *et al.* Structural insight into the mode of action of a direct inhibitor of coregulator binding to the thyroid hormone receptor. *Mol. Endocrinol.* **21**, 2919–2928 (2007).
30. Christodoulou, M. S. *et al.* Imidazo[2,1-b]benzothiazol Derivatives as Potential Allosteric Inhibitors of the Glucocorticoid Receptor. *ACS Med. Chem. Lett.* **9**, 339–344 (2018).
31. Zhou, H.-B., Collins, M. L., Gunther, J. R., Comninos, J. S. & Katzenellenbogen, J. A. Bicyclo[2.2.2]octanes: close structural mimics of the nuclear receptor-binding motif of steroid receptor coactivators. *Bioorg. Med. Chem. Lett.* **17**, 4118–4122 (2007).
32. Ravindranathan, P. *et al.* Peptidomimetic targeting of critical androgen receptor-coregulator interactions in prostate cancer. *Nat. Commun.* **4**, (2013).
33. Liu, Y. *et al.* Structural Based Screening of Antiandrogen Targeting Activation Function-2 Binding Site . *Frontiers in Pharmacology* **9**, 1419 (2018).
34. Singh, K. *et al.* Benzothiophenone derivatives targeting mutant forms of estrogen receptor-α in hormone-resistant breast cancers. *Int. J. Mol. Sci.* **19**, (2018).
35. Joseph, J. D. *et al.* Inhibition of prostate cancer cell growth by second-site androgen receptor antagonists. *Proc. Natl. Acad. Sci. U. S. A.* **106**, 12178–12183 (2009).
